# Seasonal variation in immune-related adverse events in advanced cancer patients

**DOI:** 10.1007/s00262-026-04423-x

**Published:** 2026-05-12

**Authors:** Roger Liang, Bo Gao, Adnan Nagrial, Tania Moujaber, Matteo S. Carlino, Pei N. Ding, Howard Gurney, Mark Wong, Rosemary Habib, Serigne N. Lo, Janet McKeown, Ines Pires da Silva

**Affiliations:** 1 Faculty of Medicine and Health, The University of Sydney, Camperdown, Sydney, NSW 2050 Australia; 2 Crown Princess Mary Cancer Centre, Westmead Hospital, Sydney, NSW Australia; 3 Blacktown Cancer & Haematology Centre, Blacktown Hospital, Sydney, NSW Australia; 4 Westmead Clinical School, The University of Sydney, Sydney, NSW Australia; 5 Melanoma Institute Australia, The University of Sydney, Sydney, NSW Australia; 6 Charles Perkins Centre, The University of Sydney, Sydney, NSW Australia; 7 Champalimaud Foundation, Lisbon, Portugal

**Keywords:** Seasonal, Immunotherapy, Immune checkpoint inhibitors, Immune-related adverse events, Toxicity, Advanced cancer

## Abstract

**Background:**

Immune checkpoint inhibitors (ICIs) improve outcomes across advanced cancers but can cause immune-related adverse events (irAEs). Seasonal environmental factors influence immune regulation and autoimmunity, yet its relationship with irAE development in non-melanoma cancers remains poorly defined.

**Methods:**

We retrospectively analysed patients with advanced non-melanoma solid tumours treated with anti-PD-1/PD-L1 and/or anti-CTLA-4 monotherapy at two Australian tertiary centers between 2014–2024. Seasonal variation in irAE incidence, organ distribution, severity, and timing were evaluated using descriptive analyses, Kaplan–Meier estimates and Cox proportional hazards models.

**Results:**

Among 709 patients across 27 cancer types (median follow-up 12.7 months), 514 irAEs occurred, including 32% grade ≥ 3 events and 14% treatment discontinuation rate due to toxicity. Winter ICI initiation led to the highest irAE rate (0.89 events per treatment) versus summer (0.62), largely driven by seasonal variation in low-grade cutaneous and thyroid events. Summer ICI initiation showed the highest proportion of grade ≥ 3 irAEs, whereas winter had the lowest. Seasonal effects also differed by regimen intensity, with disproportionately more irAEs among patients commencing doublet ICIs in winter. Season of ICI initiation was not significantly associated with overall irAE risk (p = 0.174) or time to onset (p = 0.11). However, time to first irAE differed by season of toxicity onset (p = 0.019), with earlier irAEs occurring in spring and summer.

**Conclusions:**

Although there was no significant association between season of ICI initiation and overall irAE risk, we observed seasonal variations in the phenotype, severity, and timing of irAEs. These exploratory findings may inform future investigation into seasonal influences on immune toxicity.

**Supplementary Information:**

The online version contains supplementary material available at 10.1007/s00262-026-04423-x.

## Introduction

Immune checkpoint inhibitors (ICIs) are an important pillar of the anticancer armamentarium for many advanced cancers, resulting in improved survival rates for patients across multiple solid tumour types [1–6]. As ICIs induce antitumour immune responses, there is a risk of generating an undesirable immune response against normal tissues. This can potentially lead to an idiosyncratic class of side effects known as immune-related adverse events (irAEs), which can affect any organ and ranges in severity from mild to life-threatening [7]. Although most irAEs are typically reversible, they can significantly impact patient quality of life from treatment interruptions, invasive diagnostic procedures, hospitalisations, and, in rare cases, permanent organ dysfunction or even death [8–10].

Known risk factors for developing irAEs with ICIs can be classified into three broad categories [11, 12]: (a) patient-related (e.g. age [13], performance status, smoking [14], history of autoimmune disease [15], vitamin D deficiency [16]), (b) malignancy-related (e.g. metastatic burden [17], tumour mutational burden [18], cancer type [19]), (c) agent-related (e.g. dose and type of ICI used [19–21], time of administration [22]). There is growing evidence supporting the impact of seasonal variation on autoimmunity, through its effects on vitamin D and melatonin levels, ultraviolet radiation exposure, and viral infections [23]. These environmental factors may modify the mechanism of epitope spreading, which is a critical mechanism underlying enhanced antitumour immune response but may also lead to loss of self-tolerance predisposing to irAE development [24].

It is important to understand the relationship between seasonality and irAEs so that underlying season-specific factors and patterns can be identified. Previous studies have demonstrated seasonal patterns of irAEs in real-world melanoma patients treated with ICIs [25–27]. There is an absence of research in this field for patients treated with ICIs for other cancer types. Hence, this study aims to analyse seasonal variability of irAE development in a real-world cohort of ICI recipients with advanced cancers other than melanoma.

## Methods

### Study design and population

This study retrospectively analysed data from patients with advanced solid tumours at two tertiary oncology centers in Sydney, Australia. We identified patients aged at least 18 years who underwent treatment with ICIs for locally advanced or metastatic cancers of any type except melanoma. ICI regimens included anti-PD-1, anti-PD-L1 or anti-CTLA-4 antibodies, either as monotherapy or in combination. We excluded regimens with concurrent administration of other systemic agents (such as chemotherapy or targeted therapy) or if ICI therapy followed induction or definitive therapy with other systemic agents (such as maintenance ICI after induction chemo-immunotherapy or consolidation ICI after definitive chemoradiation) to minimise extrinsic confounders of potential seasonal irAE signals. Adjuvant or neoadjuvant ICI regimens were also excluded, as patient characteristics and toxicity profiles in curative settings differ significantly from those in the advanced disease context. Participants must have received at least one cycle of ICI/s between January 1, 2014 and August 31, 2024, either as standard-of-care treatment or through clinical trial. The database lock was set on August 31, 2025, with all participants having at least 12 months of follow-up.

### Study procedures

Clinical data of eligible patients was collected from electronic medical records using a standardised data collection framework and managed using REDCap electronic data capture tools hosted at Western Sydney Local Health District, as approved by the local ethics committee (2024/ETH00514). Collected data included baseline patient characteristics and details of disease, treatment and outcomes. Trained clinicians retrieved data on the irAE type (grouped according to organ system), date of debut, and grade defined by Common Terminology Criteria for Adverse Events (CTCAE) version 5.0 [28]. IrAE ascertainment was based on clinician-documented diagnoses recorded in electronic medical records and, where applicable, clinical trial adverse event logs. Toxicities had to be specifically attributed to immunotherapy by the treating clinician at time of documentation to qualify for analysis. Ambiguous cases with potential alternative aetiologies were adjudicated through consensus review involving senior clinician. For immune-related endocrinologic toxicities, hepatitis, nephritis, and elevated lipase or amylase, events were included only if supported by documented abnormal laboratory parameters in addition to a clinical diagnosis and attribution to immunotherapy. For patients with multiple episodes of the same irAE, we recorded the date of debut as the first episode and the highest CTCAE grade observed across all episodes, while time-to-event analyses were conducted using the first recorded irAE per patient. Given the retrospective nature of the study, data reviewers were not blinded to the study hypotheses.

The date of the first cycle of immunotherapy and the incidence of irAEs were grouped by seasons. We defined seasons by meteorological conventions based on the annual temperature cycle for the southern hemisphere. Specifically, summer was defined as December/January/February, autumn as March/April/May, winter as June/July/August, and spring as September/October/November. There were two approaches we employed to evaluate the seasonality of irAE, based on seasonal reference points. The primary analysis assessed the incidence of irAEs according to the season of immunotherapy initiation. A secondary exploratory analysis classified irAEs according to the season of onset, defined as the season in which the irAE was first recorded. We used season of immunotherapy initiation as the primary reference point to avoid temporal bias arising from variable latency between treatment exposure and irAE development, thus enhancing the interpretability and clinical relevance of this seasonal classification.

During the preparation of this manuscript, the authors used ChatGPT 5.2 to improve readability. After using this tool, the authors reviewed and edited the content as needed and take full responsibility for the content of the published article.

### Statistical analyses

Statistical analyses were performed using R Studio, version 4.5.1 (R Core Team, Vienna, Austria), and SAS version 9.4 (SAS Institute). Baseline patient characteristics were summarised descriptively and stratified by irAE event (none versus at least one) and by season of treatment start date. Chi-squared and Wilcoxon tests were used as appropriate to assess differences between groups. Heatmaps displaying irAE frequency were generated to assess seasonal patterns in irAEs, stratified by clinical categorical variables including irAE type, irAE grade and ICI regimen. Descriptive summaries of earlier and later development of irAEs were provided using the 25th, 50th, and 75th percentiles of time to irAE, defined as date of treatment start to date of first irAE, in months. All descriptive analyses were conducted at the irAE event level, allowing patients to contribute multiple events in order to more comprehensively characterise the heterogeneity and patterns of toxicity. The Kaplan Meier method was used to analyse timing of first irAE, stratified by season of treatment initiation, and survival distributions were compared using the log-rank test. This analysis was conducted at the patient level, with the outcome defined as time to first irAE, consistent with standard time-to-event methods. A sensitivity analysis was conducted treating death or disease progression as competing events to irAE occurrence. Accordingly, a competing risks analysis was performed using cumulative incidence functions. The cumulative incidence of first irAE and of death or disease progression (competing events) were estimated and stratified by season of immunotherapy initiation, and differences between groups were assessed using Gray’s test. The Cox proportional hazards assumption was assessed for seasonality using the Schoenfeld residuals test. Univariable and multivariable Cox proportional hazards model were then used to identify factors associated with the risk of irAE occurrence. A two-sided p-value of < 0.05 was considered statistically significant. Patients with missing baseline covariate data were excluded from time-to-event and regression analyses. For descriptive summaries, patients were excluded only from analyses involving variables for which the data was missing.

## Results

### Patient characteristics

Altogether, 709 patients were included with a median age of 68 (range 18–93) and majority being male (N = 428, 60%). The most common cancer type was non-small cell lung cancer (NSCLC) in 257 (38%) patients, followed by renal cell cancer in 80 (11%) patients and endometrial cancer in 57 (8%) patients. In total, 27 cancer types were represented in this study (Supplementary Table 1). Most patients were treated for metastatic disease (N = 641, 90%), while the remaining 68 (10%) patients had locally advanced or unresectable disease. More patients received single ICI (N = 493, 70%) than doublet ICI therapy (N = 216, 30%). Similar numbers of patients received immunotherapy as first-line (N = 263, 37%) and second-line treatment (N = 325, 46%); less common were third-line (N = 95, 13%) and fourth-line or beyond (N = 26, 3%) treatment. Median follow-up time was 12.7 months (range, 0.1–132.7). Baseline patient characteristics, including subgroups stratified by irAE experienced or not, are summarised in Table 1. Patients who developed at least one irAE were more likely to have an ECOG performance status of 0 (p < 0.001) and to have received doublet ICI therapy (p < 0.001). The incidence of irAEs also differed significantly across cancer types grouped by organ system (global p < 0.001), with higher proportions observed among patients with gynaecological (71.4%) and genitourinary (58.5%) cancers. Treatment duration was longer among patients who developed irAEs (mean 11.5 vs. 6.2 months, p < 0.001), reflecting the time-dependent nature of toxicity occurrence.

**Table 1:** Baseline table of patient characteristics, by irAE or no irAE

Characteristics	All (N = 709)	No irAEs (N = 370)	At least 1 irAE (N = 339)	P-value
Age (years) at cycle 1				
Mean, SD	66.0 (12.3)	66.3 (12.1)	65.6 (12.5)	0.473
Median (range)	68.0 (18.0, 93.0)	68.0 (18.0, 93.0)	69.0 (20.0, 89.0)	
Sex				
Male	428 (60.4%)	235 (54.9%)	193 (45.1%)	0.074
Female	281 (39.6%)	135 (48.0%)	146 (52.0%)	
ECOG				
0	178 (26.4%)	64 (36.0%)	114 (64.0%)	<.001
1	385 (57.0%)	210 (54.5%)	175 (45.5%)	
2	100 (14.8%)	70 (70.0%)	30 (30.0%)	
3	12 (1.8%)	9 (75.0%)	3 (25.0%)	
Ethnicity				
Caucasian	500 (70.5%)	254 (50.8%)	246 (49.2%)	0.540
East Asian	42 (5.9%)	26 (61.9%)	16 (38.1%)	
Southeast Asian	81 (11.4%)	47 (58.0%)	34 (42.0%)	
Middle Eastern	41 (5.8%)	21 (51.2%)	20 (48.8%)	
Polynesian	19 (2.7%)	9 (47.4%)	10 (52.6%)	
Aboriginal/Torres Strait Islander	19 (2.7%)	11 (57.9%)	8 (42.1%)	
Hispanic	5 (0.7%)	2 (40.0%)	3 (60.0%)	
African	2 (0.3%)	0 (0.0%)	2 (100.0%)	
Smoking history				
Yes	465 (66.1%)	253 (54.4%)	212 (45.6%)	0.091
No	239 (33.9%)	114 (47.7%)	125 (52.3%)	
Cancer type grouped by organ system				
Gastrointestinal	86 (12.2%)	39 (45.3%)	47 (54.7%)	<.001
Genitourinary	130 (18.4%)	54 (41.5%)	76 (58.5%)	
Gynaecological	84 (11.9%)	24 (28.6%)	60 (71.4%)	
Head & Neck	50 (7.1%)	36 (72.0%)	14 (28.0%)	
Lung	305 (43.1%)	185 (60.7%)	120 (39.3%)	
Non-melanoma skin cancer	35 (5.0%)	21 (60.0%)	14 (40.0%)	
Unknown primary	17 (2.4%)	11 (64.7%)	6 (35.3%)	
Number of ICI				
Single ICI	493 (69.5%)	305 (61.9%)	188 (38.1%)	<.001
Doublet ICI	216 (30.5%)	65 (30.1%)	151 (69.9%)	
Line of ICI therapy				
1st	263 (37.1%)	122 (46.4%)	141 (53.6%)	0.074
2nd	325 (45.8%)	185 (56.9%)	140 (43.1%)	
3rd	95 (13.4%)	51 (53.7%)	44 (46.3%)	
4th	26 (3.7%)	12 (46.2%)	14 (53.8%)	
Treatment length (months)				
Mean, SD	8.7 (12.8)	6.2 (12.6)	11.5 (12.6)	<.001
Median (range)	3.4 (0.0, 116.1)	1.8 (0.0, 116.1)	6.7 (0.0, 106.4)	
Number of irAEs				
0	370 (52.2%)	370 (100.0%)	0 (0.0%)	<.001
1	216 (30.5%)	0 (0.0%)	216 (100.0%)	
2	86 (12.1%)	0 (0.0%)	86 (100.0%)	
3	25 (3.5%)	0 (0.0%)	25 (100.0%)	
4	10 (1.4%)	0 (0.0%)	10 (100.0%)	
5	1 (0.1%)	0 (0.0%)	1 (100.0%)	
6	1 (0.1%)	0 (0.0%)	1 (100.0%)	

### Immune-related adverse events

A total of 514 irAEs occurred in 339 out of 709 (48%) patients (Table 2). There were 376 (68%) mild irAEs (grades 1–2) and 138 (32%) severe irAEs (grade ≥ 3). The most frequently observed irAE subtypes were skin (N = 160/514, 31%), thyroiditis (N = 103/514, 20%) and colitis (N = 43/514, 8%) (Supplementary Fig. 1). Skin was the most common organ class affected by mild irAE with 140 (27%) occurrences, followed by thyroid (N = 98/514, 19%) (Supplementary Fig. 2). The most common severe irAE was hepatitis (N = 27/514, 5%), followed by colitis (N = 23/514, 4%) and skin (N = 20/514, 4%) (Supplementary Fig. 3). Fatal toxicities occurred in two (0.3%) patients due to pneumonitis and myocarditis.

**Table 2:** Baseline table for irAE events by season of immunotherapy treatment start

Characteristics	All (N = 514)	Autumn (N = 125)	Winter (N = 161)	Spring (N = 135)	Summer (N = 93)	P-value
Time from first immunotherapy to first irAE						
Early	193 (37.5%)	57 (29.5%)	48 (24.9%)	52 (26.9%)	36 (18.7%)	0.044
Intermediate	294 (57.2%)	64 (21.8%)	106 (36.1%)	76 (25.9%)	48 (16.3%)	
Late	27 (5.3%)	4 (14.8%)	7 (25.9%)	7 (25.9%)	9 (33.3%)	
Time from first immunotherapy to first irAE (quartiles)						
1 st (0–0.6 months)	161 (31.3%)	48 (29.8%)	40 (24.8%)	42 (26.1%)	31 (19.3%)	0.126
2nd (0.7–1.5 months)	117 (22.8%)	31 (26.5%)	35 (29.9%)	31 (26.5%)	20 (17.1%)	
3rd (1.6–4.09 months)	132 (25.7%)	31 (23.5%)	47 (35.6%)	36 (27.3%)	18 (13.6%)	
4th (4.10 months and over)	104 (20.2%)	15 (14.4%)	39 (37.5%)	26 (25.0%)	24 (23.1%)	
irAE grade						
1	143 (27.8%)	40 (28.0%)	46 (32.2%)	32 (22.4%)	25 (17.5%)	0.219
2	233 (45.3%)	55 (23.6%)	77 (33.0%)	67 (28.8%)	34 (14.6%)	
3	124 (24.1%)	28 (22.6%)	33 (26.6%)	32 (25.8%)	31 (25.0%)	
4	12 (2.3%)	2 (16.7%)	5 (41.7%)	2 (16.7%)	3 (25.0%)	
5	2 (0.4%)	0 (0.0%)	0 (0.0%)	2 (100.0%)	0 (0.0%)	
Tumour type grouped by organ system						
Gastrointestinal	71 (13.9%)	18 (25.4%)	21 (29.6%)	14 (19.7%)	18 (25.4%)	0.022
Genitourinary	111 (21.8%)	36 (32.4%)	28 (25.2%)	32 (28.8%)	15 (13.5%)	
Gynaecological	105 (20.6%)	17 (16.2%)	45 (42.9%)	29 (27.6%)	14 (13.3%)	
Head & Neck	18 (3.5%)	2 (11.1%)	6 (33.3%)	4 (22.2%)	6 (33.3%)	
Lung	177 (34.8%)	40 (22.6%)	53 (29.9%)	48 (27.1%)	36 (20.3%)	
Non-melanoma skin cancer	14 (2.8%)	5 (35.7%)	6 (42.9%)	2 (14.3%)	1 (7.1%)	
Unknown primary	13 (2.6%)	4 (30.8%)	0 (0.0%)	6 (46.2%)	3 (23.1%)	
ECOG						
0	195 (40.0%)	56 (28.7%)	48 (24.6%)	60 (30.8%)	31 (15.9%)	0.151
1	248 (50.9%)	51 (20.6%)	90 (36.3%)	61 (24.6%)	46 (18.5%)	
2	38 (7.8%)	10 (26.3%)	12 (31.6%)	9 (23.7%)	7 (18.4%)	
3	6 (1.2%)	1 (16.7%)	3 (50.0%)	0 (0.0%)	2 (33.3%)	
Number of ICI						
Single ICI	254 (49.4%)	69 (27.2%)	71 (28.0%)	74 (29.1%)	40 (15.7%)	0.086
Doublet ICI	260 (50.6%)	56 (21.5%)	90 (34.6%)	61 (23.5%)	53 (20.4%)	
Treatment of irAE						
Oral corticosteroids	192 (37.4%)	43 (22.4%)	54 (28.1%)	55 (28.6%)	40 (20.8%)	0.360
Biologic agents	28 (5.4%)	5 (17.9%)	7 (25.0%)	10 (35.7%)	6 (21.4%)	
Neither	294 (57.2%)	77 (26.2%)	100 (34.0%)	70 (23.8%)	47 (16.0%)	

Multiple irAEs were experienced by 123 (17%) patients (Supplementary Table 2). Additional baseline irAE characteristics, grouped by season of ICI initiation, are presented in Table 2. There were 193 (38%) irAEs that developed ‘early’, defined as onset within a month following ICI initiation, compared to only 27 (5%) irAEs with ‘late’ onset, occurring more than 12 months after ICI initiation. The median time between treatment initiation and onset of first irAE was 1.6 months, while the median time to any irAE was 2.5 months. The maximal pharmacologic management for irAEs were oral corticosteroids for 192 (37%) toxicities and biologic immunosuppressants for 28 (5%) toxicities. In total, 101 (14%) patients discontinued immunotherapy due to irAEs.

### Seasonal distribution and risk of immune-related adverse events

**Fig. 1:**
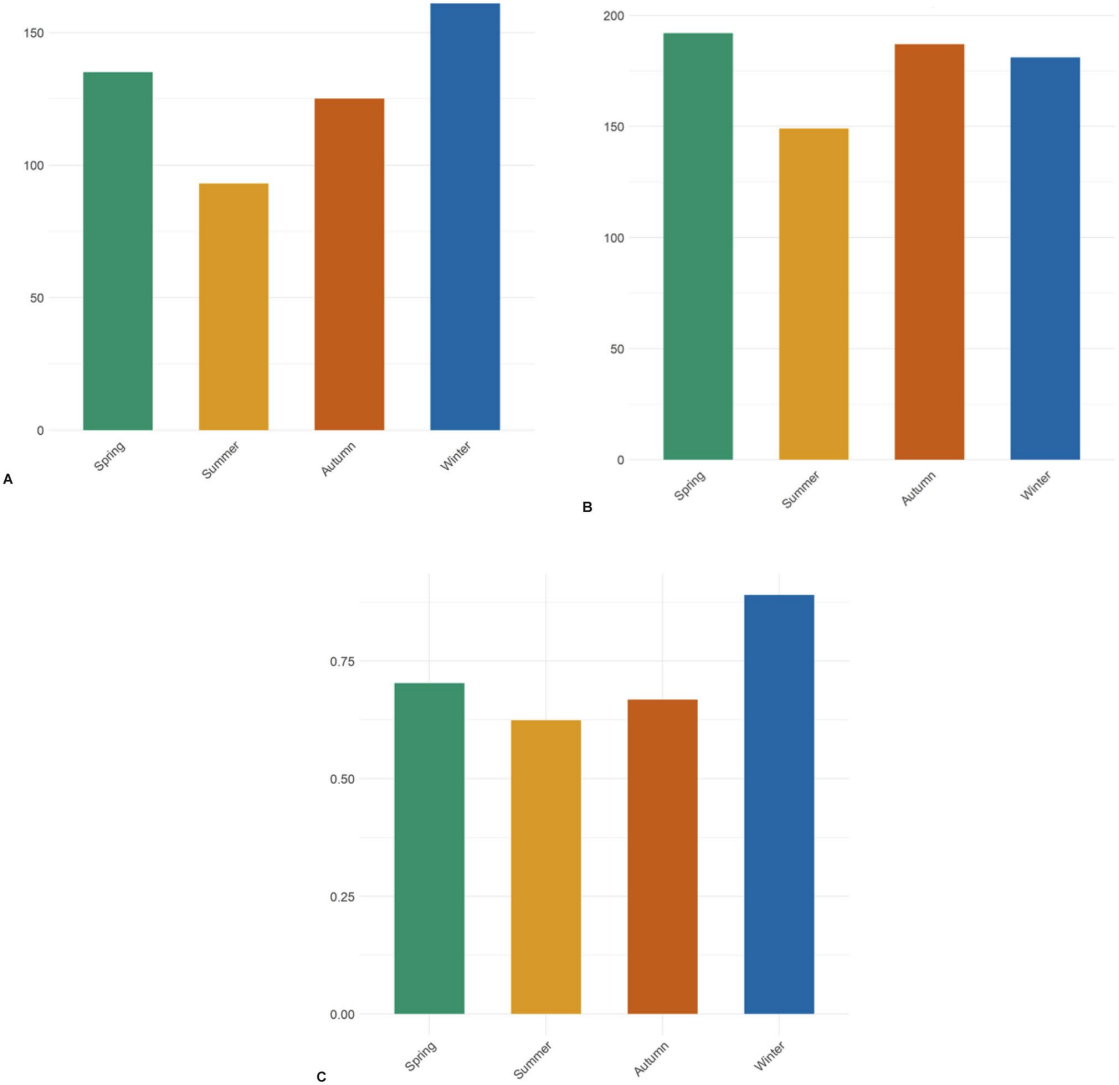
**A** Total number of irAEs by season of immunotherapy initiation. **B**. Total number of immunotherapy treatments initiated in each season. **C**. Ratio of number of irAEs to number of immunotherapy treatments initiated in each season

ICI treatment initiated in winter led to the highest number of irAEs compared to other seasons with 161 events representing 31% of total irAEs (Fig. 1A). Meanwhile, ICI treatment commenced in summer resulted in the lowest number of toxicity events (N = 93/514, 18%). However, fewer treatments were initiated in summer (N = 149/709, 21%) compared to winter, autumn or spring (N = 181, N = 187, and N = 192, respectively) (Fig. 1B). To account for this seasonal variation in treatment administration, we calculated the ratio of irAEs per ICI treatment initiated for each season (Fig. 1C). Even after this adjustment, the toxicity ratio remained highest for winter and lowest for summer (0.89 and 0.62, respectively).

Season of ICI initiation was not significantly associated with irAE occurrence in the primary multivariable Cox model (overall p = 0.174; Supplementary Table 3, Supplementary Fig. 4), and there was no evidence of violation of the proportional hazard assumptions for seasonality (Schoenfeld test p = 0.31). In a competing risks analysis, the cumulative incidence of irAE did not differ significantly by season of treatment initiation (Gray’s test p = 0.24). Similarly, there was no significant seasonal difference in the competing event of death or disease progression (p = 0.17; Supplementary Fig. 5). In a sensitivity analysis restricted to lung cancer patients (N = 305/709), findings were broadly consistent with the primary analysis, with no significant association between season of ICI initiation and irAE risk in the multivariable model (overall p = 0.5376; Supplementary Table 4).

The distribution of irAEs by season of onset was more equal, with the most events occurring in spring (N = 138/514, 27%), followed by autumn, winter, then summer (N = 128, N = 125, and N = 123, respectively).

### Seasonal patterns of immune-related adverse events

**Fig. 2:**
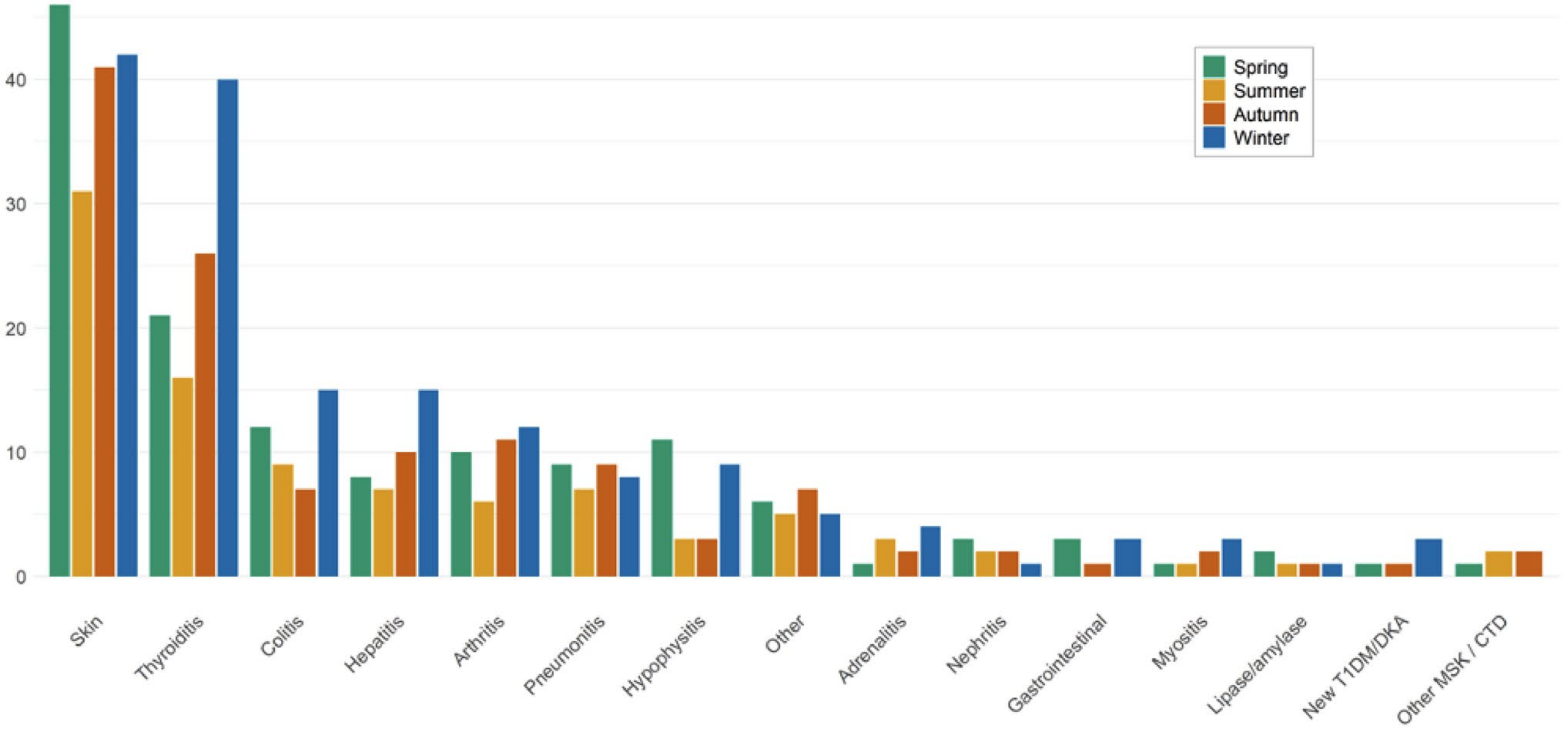
Seasonal distribution of irAEs. Number of toxicity events are shown per irAE type per season. IrAE types occurring < 5 times in total are categorised under ‘Other’, which includes cardiac (N = 4), haematological (N = 4), neurologic (N = 4), xerostomia/Sicca syndrome (N = 4), sarcoid-like immune reaction (N = 3), ocular (N = 2), pancreatitis (N = 1), pleural immune reaction (N = 1). Abbreviations: T1DM; type 1 diabetes mellitus, DKA; diabetic ketoacidosis, MSK; musculoskeletal, CTD; connective tissue disease

We evaluated whether incidence of specific irAE subtypes varied by season (Fig. 2). When stratified by season of ICI initiation, skin irAEs were least common following summer initiation (N = 31/160, 19%). Thyroid irAEs were most frequent for winter treatment initiation (N = 40/103, 39%) and least frequent with summer initiation (N = 16/103, 16%). Winter-initiated treatments also demonstrated the highest proportions of hepatitis (N = 15/40, 38%), colitis (N = 15/43, 35%) and arthritis (N = 12/39, 31%) irAEs relative to other seasons. When stratified by the season of irAE onset, summer again observed the lowest proportions of skin (N = 34/160, 21%) and hepatitis (N = 4/40, 10%) irAEs, while pneumonitis most frequently debuted in winter (N = 13/33, 39%) (Supplementary Fig. 6).

**Fig. 3:**
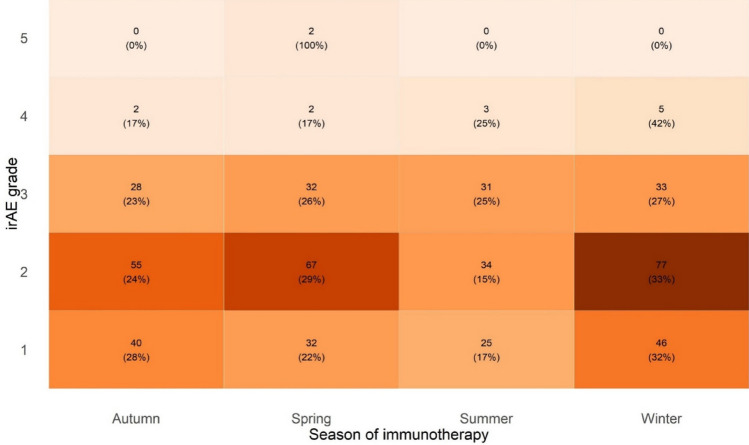
Heatmap of irAEs by grade against season of start of immunotherapy

Season of ICI treatment initiation was associated with irAE severity, with summer associated with the highest proportion of grade ≥ 3 irAEs at 37% (N = 34/93), and winter associated with the lowest proportion of grade ≥ 3 irAEs at 24% (N = 38/161) (Fig. 3). No meaningful differences in the proportion of higher-grade irAEs were observed when analyses were stratified by the season of irAE onset (Supplementary Fig. 7).

**Fig. 4:**
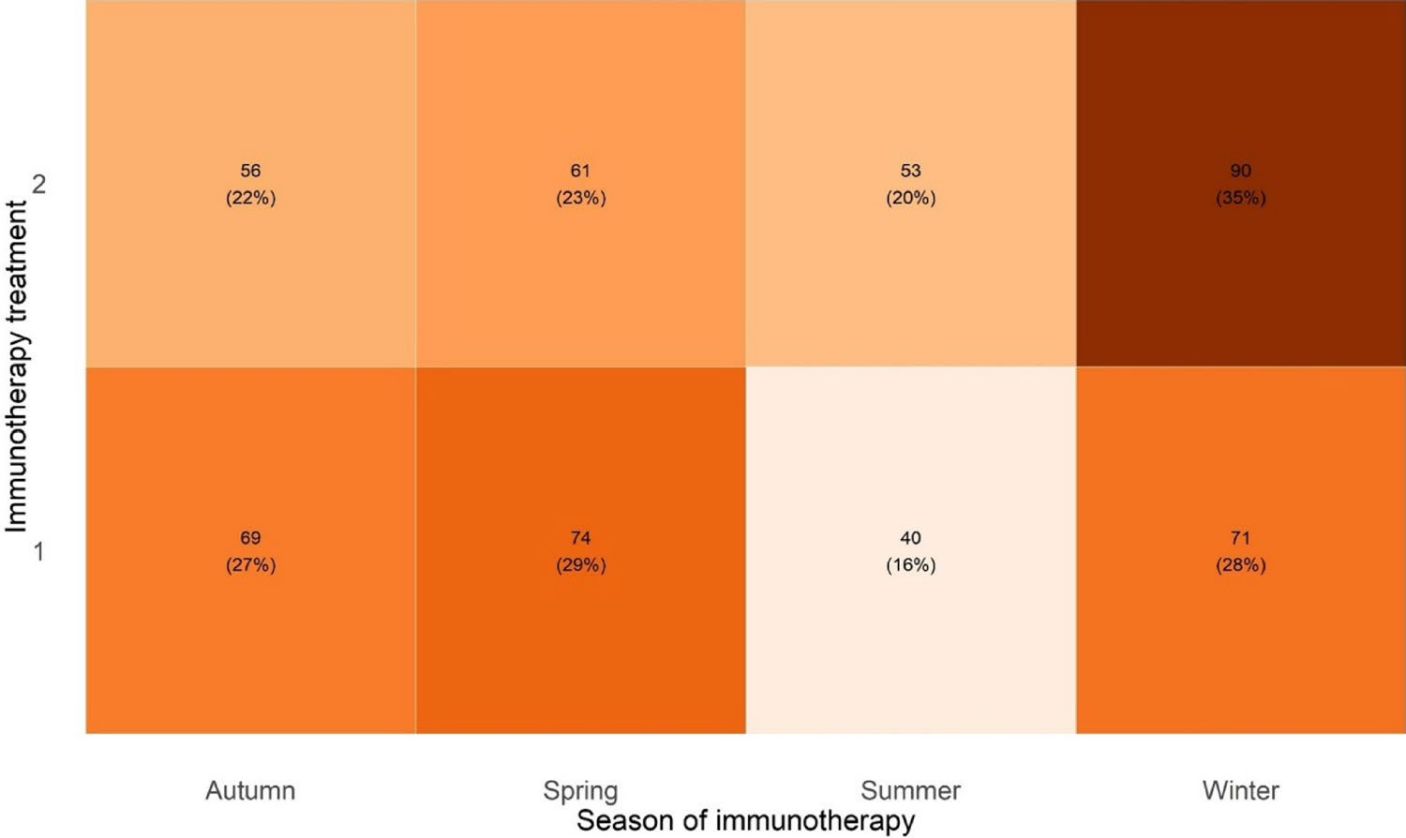
Heatmap of irAEs by number of ICIs in treatment regimen (1 = single agent ICI, 2 = doublet ICIs) against season of start of immunotherapy

A seasonal influence on irAEs was observed according to the number of ICIs administered (Fig. 4). Single agent ICI treatments initiated in summer were associated with fewer irAEs (N = 40/254, 16%) compared with other seasons, whereas doublet ICI regimens initiated in winter were associated with a disproportionately higher incidence of irAEs (N = 90/260, 35%).

### Seasonal influence on time to onset of immune-related adverse events

**Fig. 5:**
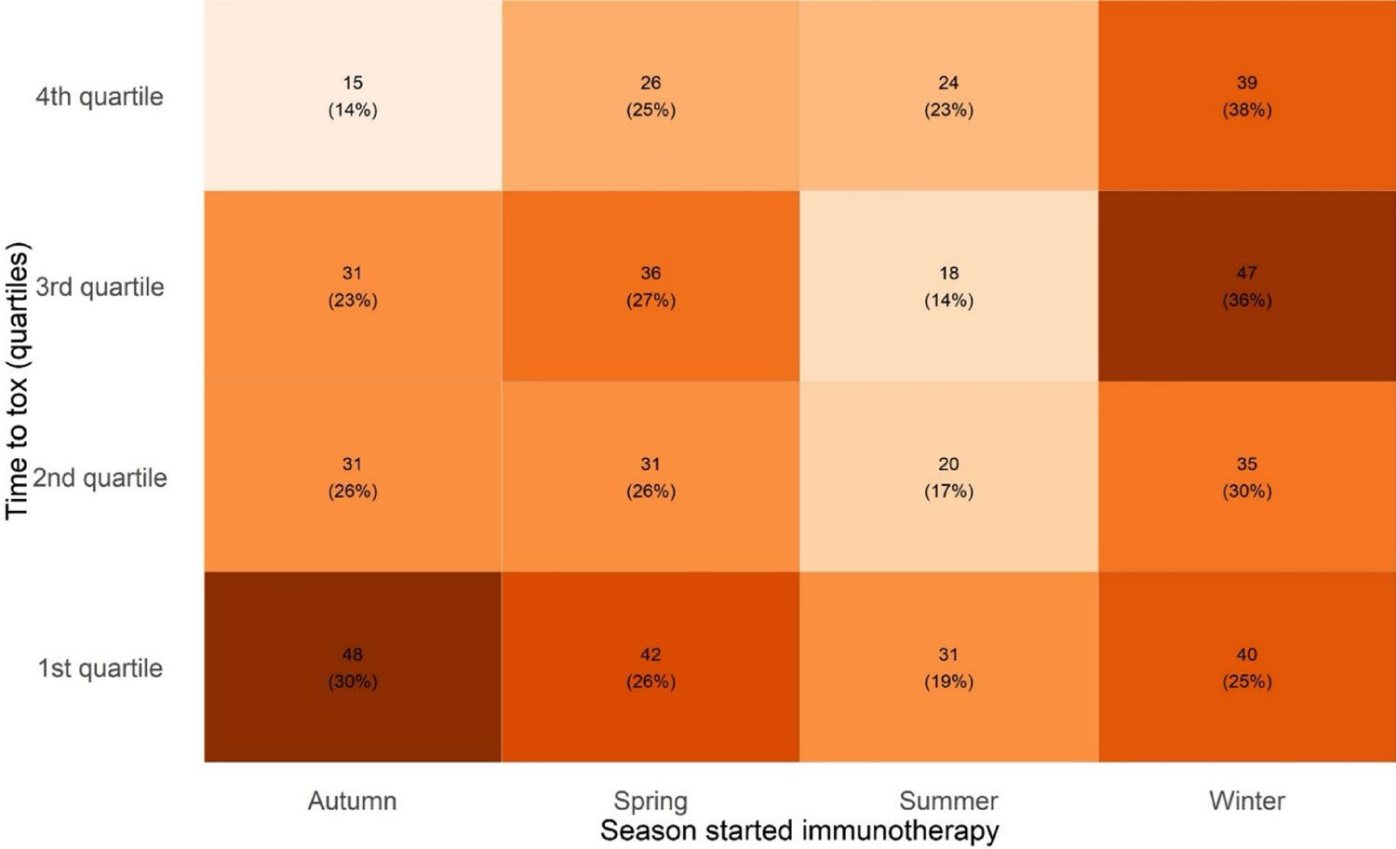
Heatmap of time to irAE from start of immunotherapy, divided into quartiles (1st = 0 to 0.6 months; 2nd = 0.7 to 1.5 months; 3rd = 1.6 to 4.09 months; 4th = 4.10 months and over), against season of start of immunotherapy

The timing of irAE onset, stratified by time quartiles, varied by the season of immunotherapy initiation (Fig. 5). Autumn-initiated patients who experienced an irAE showed a relative shift towards early-quartile events, where 38% (N = 48/125) of irAEs occurred in the first quartile (less than 0.7 months) compared to only 12% (N = 15/125) occurring in the fourth quartile (more than 4.09 months). In contrast, winter initiation was associated with a higher proportion of late-quartile irAEs, where 53% (N = 86/161) of winter-initiated patients with irAE developed toxicity in the third and fourth quartiles (more than 1.5 months). Spring- and summer-initiated patients demonstrated a more balanced distribution of irAE timing across quartiles. When analysed by season of irAE onset, similar early-quartile trends were observed for autumn, while summer saw relatively more late-quartile irAEs (Supplementary Fig. 8).

**Fig. 6:**
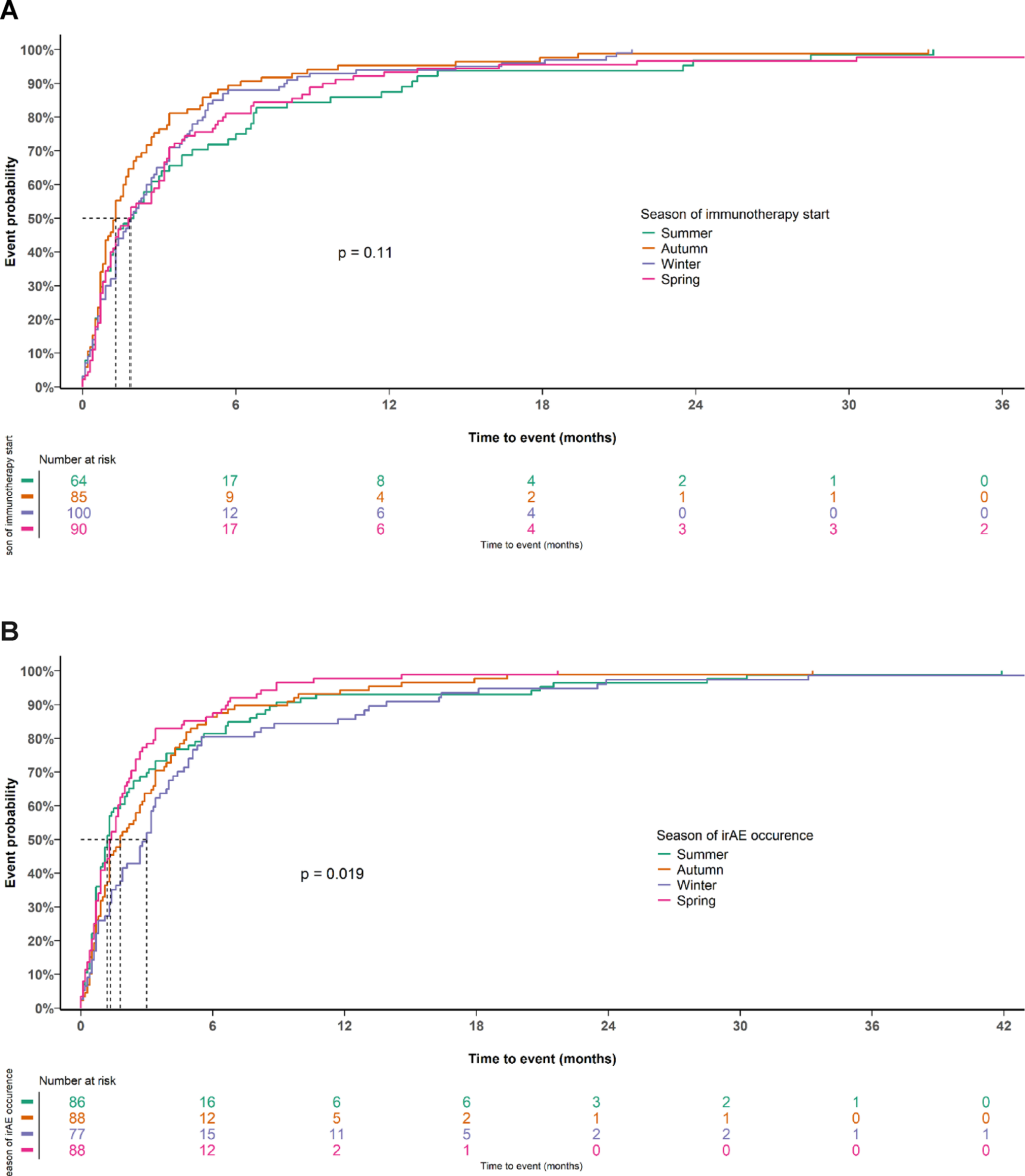
**A** Kaplan–Meier estimate of time to first irAE stratified by season of immunotherapy initiation for all patients with an irAE. **B** Kaplan–Meier estimate of time to first irAE stratified by season of irAE onset for all patients with an irAE

Kaplan–Meier analysis was used to evaluate whether there was a seasonal influence on time to development of irAEs. When stratified by season of immunotherapy initiation, there were no significant differences in time to irAE onset across seasons (p = 0.11; Fig. 6A). Similarly, there were no seasonal differences in time to onset of common irAEs like skin, thyroiditis, colitis, hepatitis, and arthritis (Supplementary Fig. 9A-E). Analysis was also stratified according to the season of irAE onset, reflecting our hypothesis that seasonal environmental exposures are more likely to influence the timing of development of various organ-specific toxicities than the season of immunotherapy initiation. This demonstrated that time to first irAE differed significantly by season (p = 0.019; Fig. 6B), with events occurring in spring and summer arising earlier after treatment initiation.

## Discussion

In this real-world cohort of patients with multiple types of advanced cancers treated with ICIs, there was no significant association between season of ICI treatment initiation and overall irAE occurrence, with consistent results across multivariable regression, time-to-event, and competing risks analyses. However, we observed descriptive seasonal variation in some toxicity patterns, including incidence, severity, timing, and subtype. ICI treatment initiated in winter was associated with the highest overall incidence of irAEs, driven predominantly by common, low-grade toxicities, such as skin and thyroiditis, with a disproportionate contribution from doublet ICI regimens started during this season. In contrast, summer initiation was associated with the lowest irAE incidence, especially with single agent ICI regimens, despite having the highest proportion of grade ≥ 3 irAEs. Additionally, we identified a signal of seasonal variation in the timing of onset of irAEs, for example, winter was associated with the development of later onset toxicities.

Previous retrospective studies involving patients undergoing ICI therapy for various indications have indicated the influence of seasonality on irAEs. In a Danish study of resected melanoma patients receiving adjuvant anti-PD-1 ICI therapy [25], cutaneous irAEs, which were mainly grade 1, were seen more frequently in the winter-autumn seasons, while the incidence of grade 2–5 irAEs was highest in the summer-spring seasons. Similarly, cutaneous irAE incidence peaked in patients initiating treatment in winter in a large multicenter cohort of ICI recipients in the United States [26]. In their analysis of 394 melanoma patients treated at centers in both the northern and southern hemispheres, Rogiers et al. [27] demonstrated the treatment-adjusted incidence of irAEs was highest in winter and lowest in summer. Our study reinforces the emerging literature on seasonality, as we also demonstrate that winter was associated with the highest irAE incidence, mainly driven by higher rates of low-grade cutaneous and thyroiditis toxicities, while the lowest frequencies of these irAE subtypes were observed with summer-initiated treatment. These observations may be considered in the context of prior studies describing seasonal variation in immune cell profiles and gene expression of inflammatory proteins in the circulation, which exhibit a circannual rhythm of pro-inflammatory activation in winter and relative immunosuppression in summer [29, 30]. However, these biological factors were not assessed in the current study, and their relevance to our observed seasonal patterns remains uncertain. Similarly, the apparent increase in cutaneous irAEs observed in winter may reflect climate-related skin barrier dysfunction and greater exposure to respiratory viruses contributing to autoimmune susceptibility [31, 32], although this also remains speculative. Circannual variations in thyroid axis physiology have been described, including seasonal fluctuations in serum TSH levels and an association between winter and increased risk of transition from euthyroid status to subclinical hypothyroidism [33, 34]. While our observation of an increased risk of thyroiditis with winter-initiated treatment aligns with these existing biological datasets, this potential relationship requires confirmation in prospective and translational studies.

We also found that the next three most common irAE subtypes – colitis, hepatitis, and arthritis – occurred most frequently when ICI therapy was initiated in winter. These observations align with established literature linking winter-associated reductions in cutaneous ultraviolet (UV) exposure and serum vitamin D levels to increased autoimmune disease activity and relapse risk [23]. UV radiation exerts systemic immunosuppressive effects independent of vitamin D status [35, 36], raising the possibility that reduced UV exposure during winter may influence susceptibility to irAEs. In addition to observational data linking vitamin D status with irAE risk [16], a recent mechanistic study showed that vitamin D modulates T helper cell function and immune plasticity [37], providing a biological basis for how seasonal variation in vitamin D availability could influence immune tolerance and the clinical expression of irAEs. While previous observational and prospective studies suggest that vitamin D supplementation may mitigate irAE risk in ICI-treated patients [38, 39], we could not assess this potential association in our cohort due to the absence of vitamin D levels and supplementation data. Therefore, whether these environmental factors independently drive seasonal irAE patterns remains a hypothesis for future investigation.

We conducted secondary exploratory analyses using the season of irAE onset as an alternative reference point to descriptively examine season-specific patterns in the phenotype and timing of irAE development. Although stratifying by season of irAE occurrence introduces potential circularity and calendar-based bias, we employed this approach as a hypothesis-generating tool to investigate whether specific toxicities appear to cluster within seasonal periods. Consistent with prior research [27], we observed that pneumonitis most frequently debuted in winter. This may reflect seasonal variation in respiratory pathogen exposure and exacerbations of underlying respiratory comorbidities, but may also be confounded by diagnostic challenges in distinguishing immune-related pneumonitis from other winter-associated respiratory conditions. We also found an exploratory signal that irAEs occurring in summer and spring developed faster after treatment initiation than those occurring in winter and autumn, suggesting that seasonality may influence the timing of irAE development. While a prior study reported seasonal patterns in faster development of various irAE subtypes [27], our analysis of individual irAEs did not demonstrate any statistically significant difference in timing. However, thyroiditis demonstrated a trend towards delayed onset when this toxicity occurred in winter (p = 0.094, Supplementary Fig. 9B). This pattern raises the possibility that permissive seasonal factors present in winter may be required for thyroiditis to manifest, leading to an apparently prolonged time to event. Although consistent with known seasonal variation in autoimmune thyroid disease [33, 34], this finding is hypothesis-generating and warrants further investigation.

Descriptive analysis of the timing of irAE onset, stratified by quartiles, showed seasonal variation in the distribution of events: autumn initiation coincided with a higher frequency of early-quartile irAEs, while winter-treated patients had a relative concentration of irAEs in later time quartiles. A possible explanation for these observations is seasonal variation in immune function, with autumn representing a transition toward the heightened baseline inflammatory activity described in winter in the biological literature [29, 30], which may influence the timing of immune-related toxicity. Alternatively, these patterns may also reflect calendar-related effects; for example, the apparent earlier onset of irAEs observed with autumn initiation may arise because early events occur during the subsequent winter period, rather than indicating a distinct seasonal effect at treatment initiation. Distinguishing between these potential biological and calendar-driven hypotheses is not possible within this retrospective study, highlighting the need for future prospective and translational research.

This study also identified descriptive differences in irAE severity and risk across seasons that appeared to vary by ICI regimen. We observed opposing severity profiles between winter, which had the lowest proportion of grade ≥ 3 irAEs but highest total number of irAEs, and summer initiation, which had the highest proportion of grade ≥ 3 irAEs and fewest overall irAEs. This pattern may reflect seasonal variation in the distribution of irAE subtypes, with cutaneous and thyroiditis toxicities that are typically lower grade being more frequently represented in winter. We also observed regimen-specific seasonal patterns. Among irAEs occurring with single agent anti–PD-1/PD-L1 therapy, a smaller proportion arose with summer initiation (16% of all irAEs for this regimen), whereas irAEs associated with doublet (anti–PD-1/PD-L1 plus anti–CTLA-4) regimens were more frequently observed following winter initiation (35% of all irAEs for this regimen). The distinct immunologic mechanisms of ICIs, such as the early priming of T-cell activation within lymphoid tissues by CTLA-4 blockade versus the modulation of peripheral effector responses with PD-1/PD-L1 inhibition [40], could interact with seasonal variation in baseline immune activity to offer a theoretical framework for these patterns. For example, in winter characterised by a pro-inflammatory immune state, initiation of anti-CTLA-4-based therapy could theoretically amplify upstream immune activation and increase the propensity for irAEs in this season. However, these mechanistic hypotheses cannot be confirmed by our descriptive data and should therefore be considered exploratory, requiring further investigation to assess whether seasonality influences regimen-specific toxicity risk.

There were several limitations to our study. The retrospective design may have affected irAE detection, with the potential for both under- and over-reporting depending on clinical documentation, as well as misattribution of toxicities to irAEs. The timing of irAE onset may also be imprecise, reflecting delays between manifestation and clinical recognition, which depend on the scheduling of clinical encounters and frequency of laboratory monitoring. Importantly, our primary analysis is limited by several unmeasured factors that could confound seasonal signals. These include calendar period effects, evolving clinical practice over the ten-year accrual period, and health system or behavioural factors impacting healthcare utilisation and clinical testing across seasons. Residual confounding may also have influenced the primary multivariable Cox model, as covariates such as tumour type were grouped by organ system to minimise model overfitting. Furthermore, the subgroup and secondary analyses involve comparisons across multiple irAE subtypes and seasonal bins without multiple-testing correction, increasing the risk of Type I error. Exclusion of combination regimens with non-ICI therapies may limit generalisability of our findings to contemporary clinical practice. Finally, as this cohort comprised patients treated in a region in Australia with relatively temperate climatic conditions, the generalisability of these findings to regions with more extreme or diverse seasonal patterns may be limited.

Notwithstanding these limitations, this study has several strengths. To our knowledge, it represents the first large real-world analysis of seasonality across the full spectrum of irAEs in ICI recipients with cancers other than melanoma. Additionally, all patients received ICI as monotherapy in the advanced disease setting, without prior neoadjuvant, adjuvant or induction combination exposure, which enables a more uniform assessment of seasonal effects on irAEs with reduced confounding. Further research with standardised irAE assessment and inclusion of cohorts from diverse geographic and climatic regions will be imperative to clarify the role of seasonality in irAEs.

In conclusion, although our primary analysis did not show a significant association between season of treatment initiation and overall irAE risk, this large real-world dataset provides valuable descriptive evidence suggesting potential seasonal variation in immunotherapy-related toxicity across a diverse patient population with multiple tumour types represented. We identified season-specific patterns in the incidence, spectrum, severity, and timing of various irAEs, as well as a consistent, albeit non-significant, signal of a higher overall burden of irAEs occurring in patients who commenced ICI therapy during winter. Collectively, these findings suggest that seasonal context may influence the clinical presentation and phenotype of irAEs, although the underlying mechanisms remain uncertain. These exploratory observations provide a rationale for future prospective and translational research to better characterise seasonal influences on immunotherapy-related toxicity.

## Supplementary Information

Below is the link to the electronic supplementary material.

Supplementary file1 (DOCX 2058 KB)

## Data Availability

The datasets analysed during the current study are available from the corresponding author on reasonable request.
