# Supplementary material for: Seasonal variation in immune-related adverse events in advanced cancer patients
Source: Cancer Immunol Immunother. 2026 May 12;75(7):180. doi: 10.1007/s00262-026-04423-x (PMC13341991; doi:10.1007/s00262-026-04423-x)
Supplement: Supplementary file 1 — Supplementary file1 (DOCX 2058 KB) [file 262_2026_4423_MOESM1_ESM.docx]

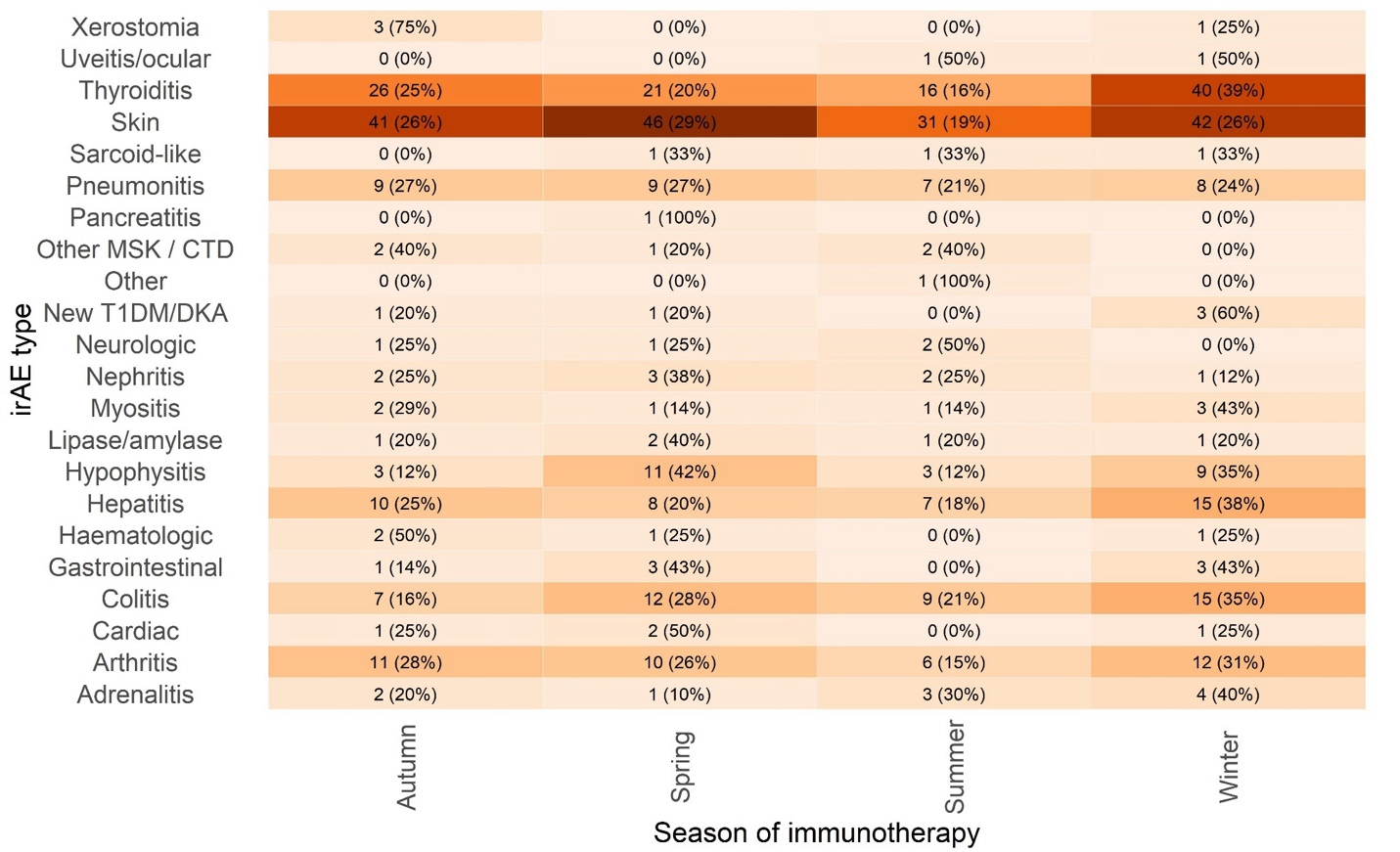


**Supplementary Figure 1.** Heatmap of type of irAE experienced, against season of start of immunotherapy.


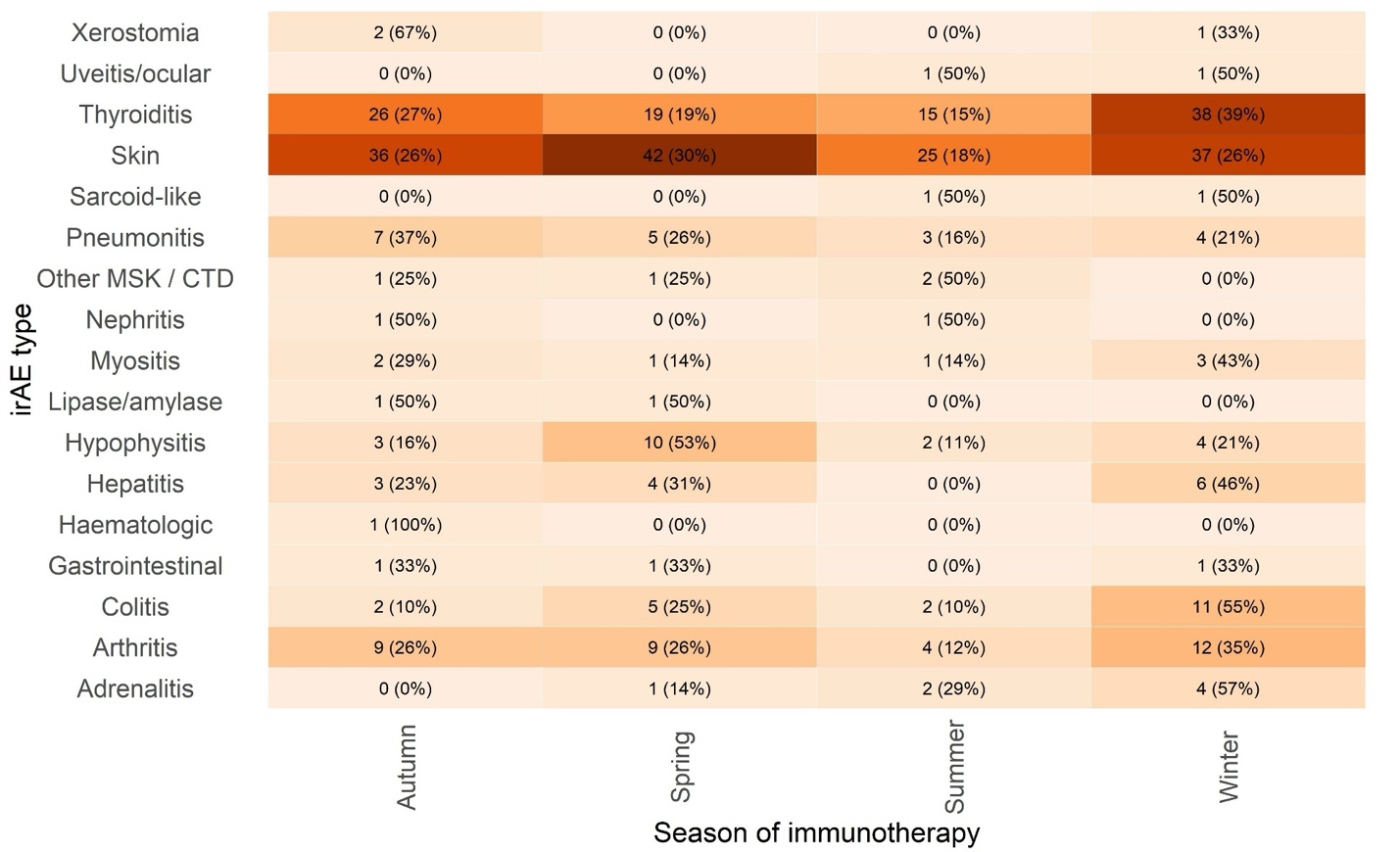
 **Supplementary Figure 2.** Heatmap of type of irAE experienced for grades 1&2, against season of start of immunotherapy.


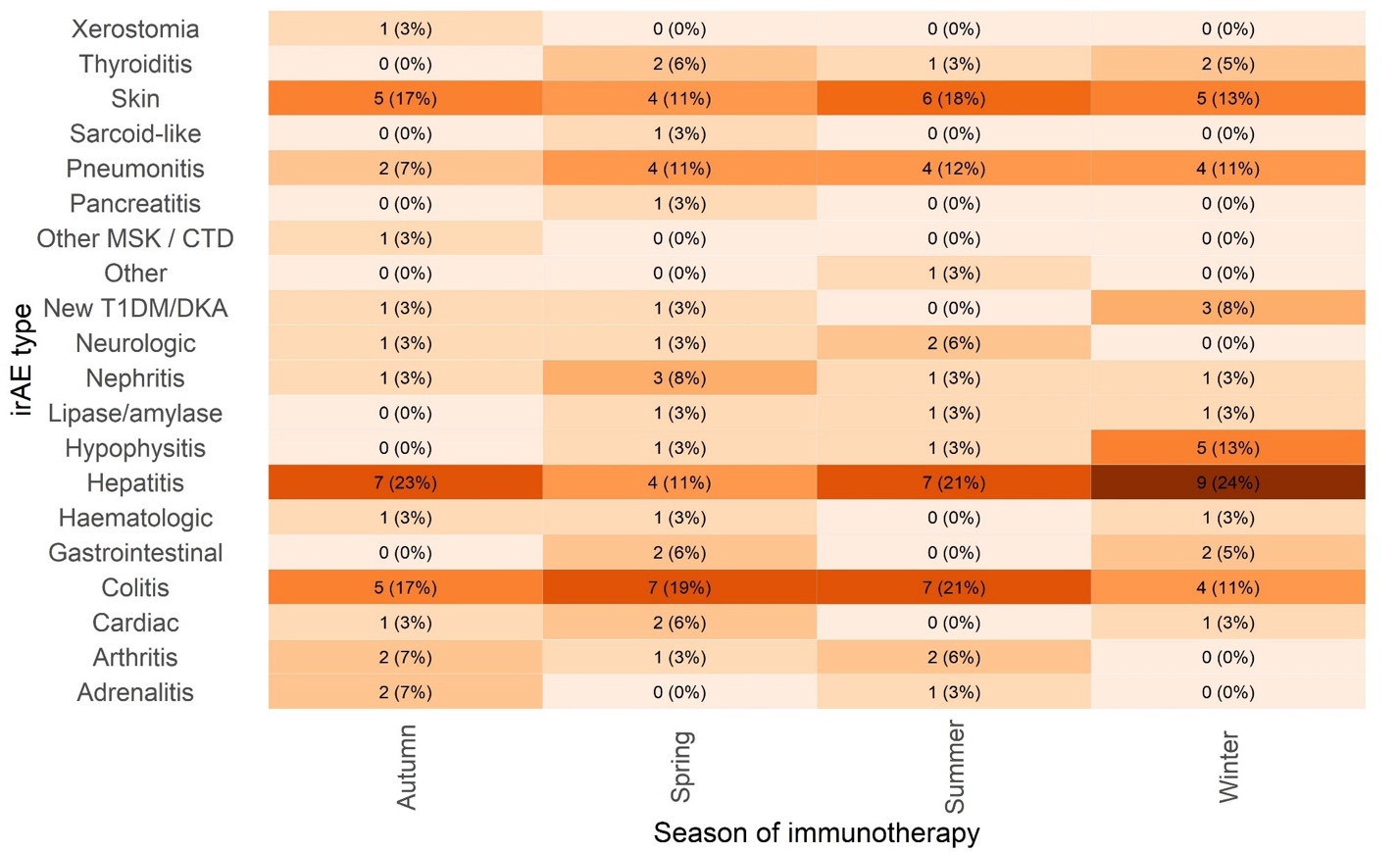


**Supplementary Figure 3.** Heatmap of type of irAE experienced for grades 3 to 5, against season of start of immunotherapy.


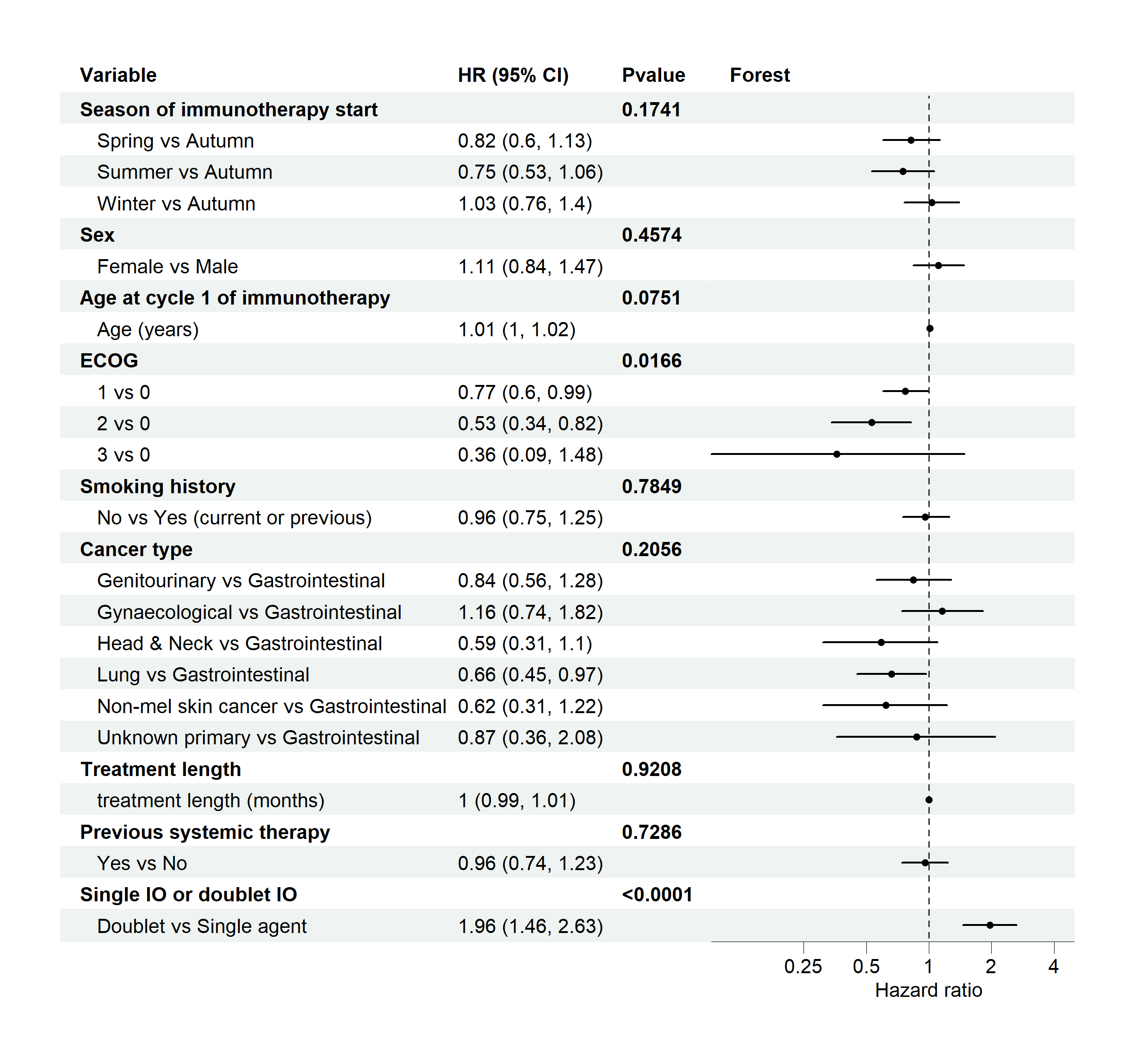


**Supplementary Figure 4.** Forest plot for multivariable Cox regression for irAE (yes vs no).


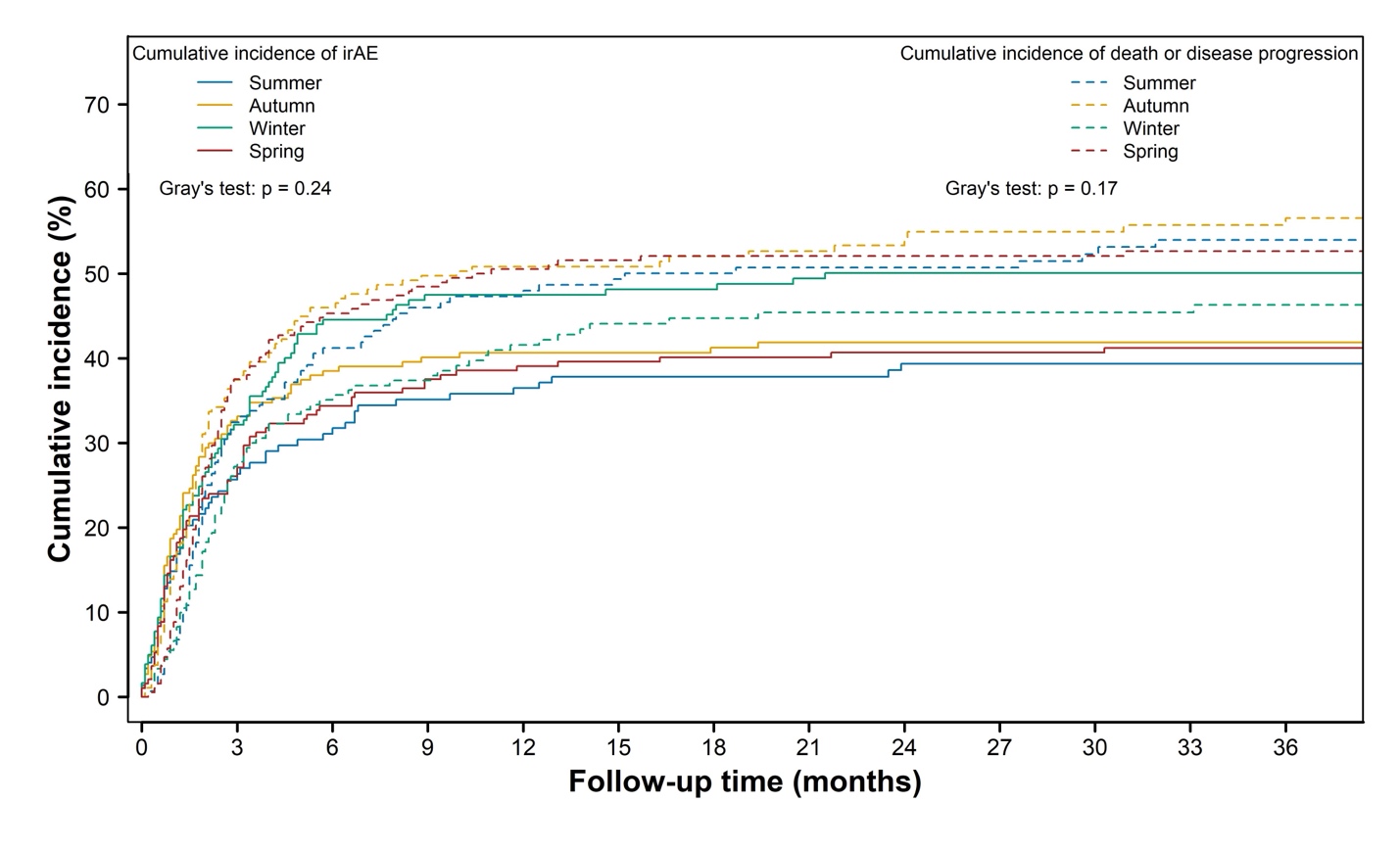


**Supplementary Figure 5**. Competing risks model comparing cumulative incidence functions for irAE vs death or disease progression.


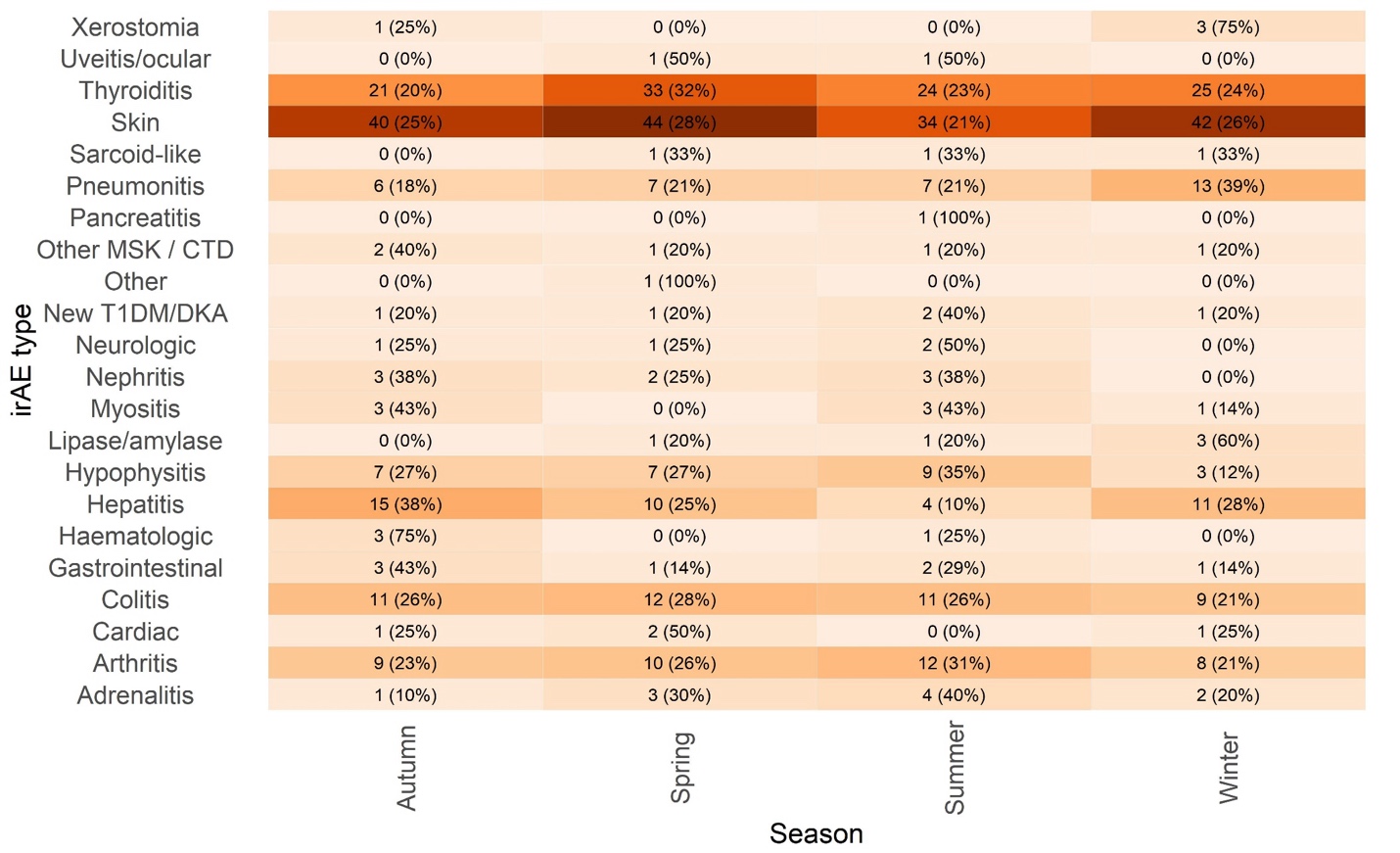


**Supplementary Figure 6**. Heatmap of type of irAE experienced, against season of irAE onset.


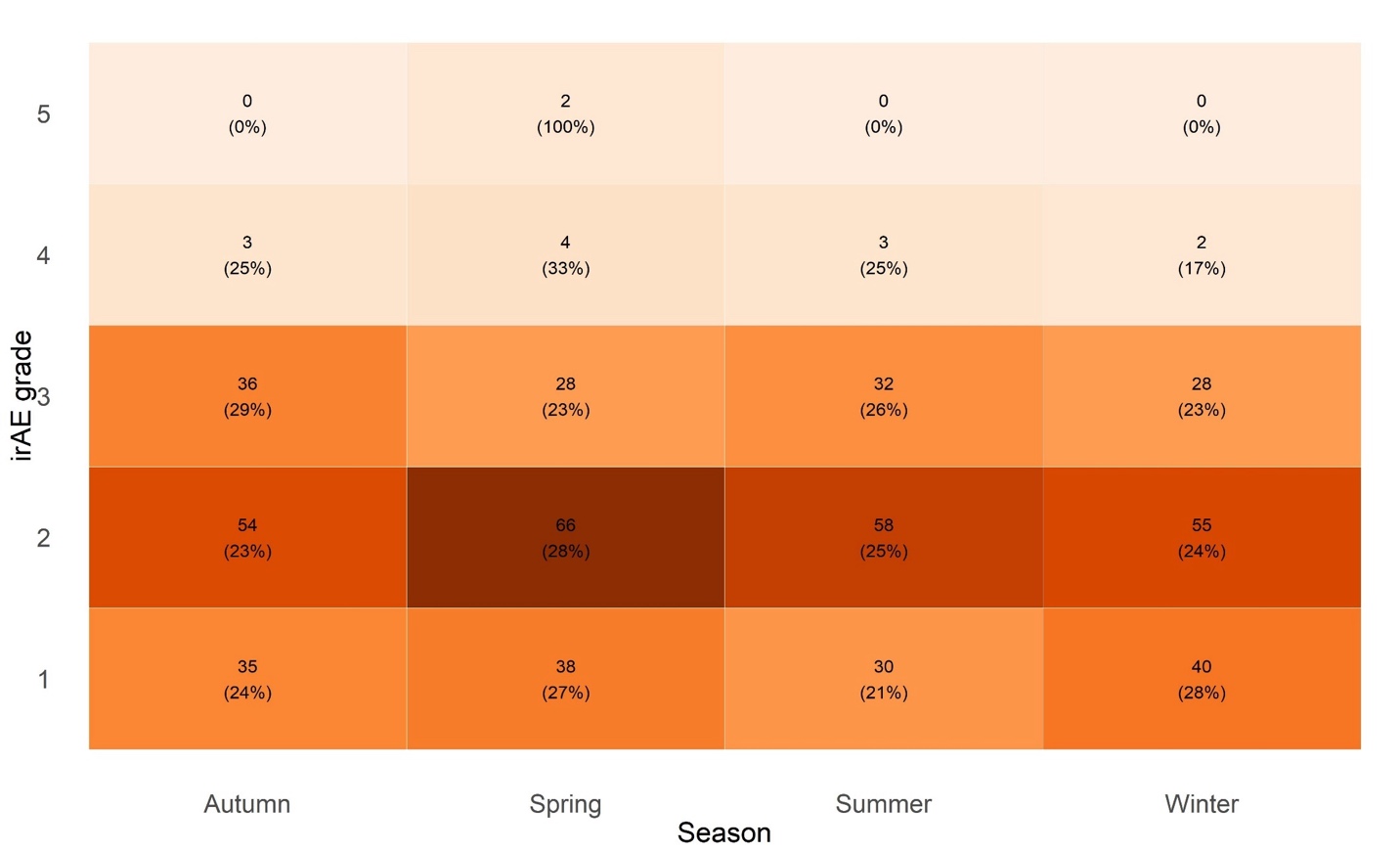


**Supplementary Figure 7**. Heatmap of irAE grade, against season of irAE onset.


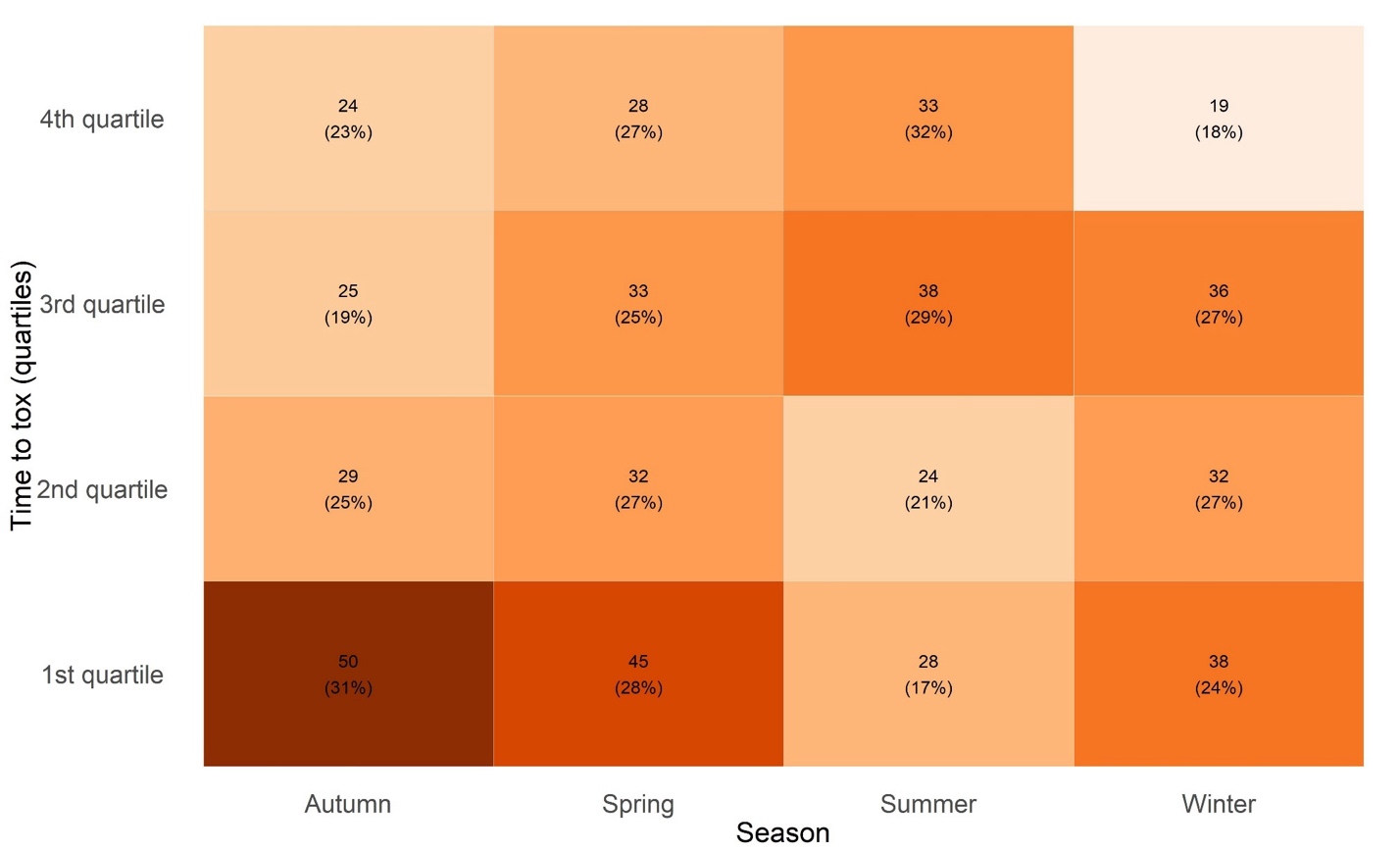


**Supplementary Figure 8**. Heatmap of time to irAE from start of immunotherapy, divided into quartiles (1st = 0 to 0.6 months; 2nd = 0.7 to 1.5 months; 3rd = 1.6 to 4.09 months; 4th = 4.10 months and over), against season of irAE onset.


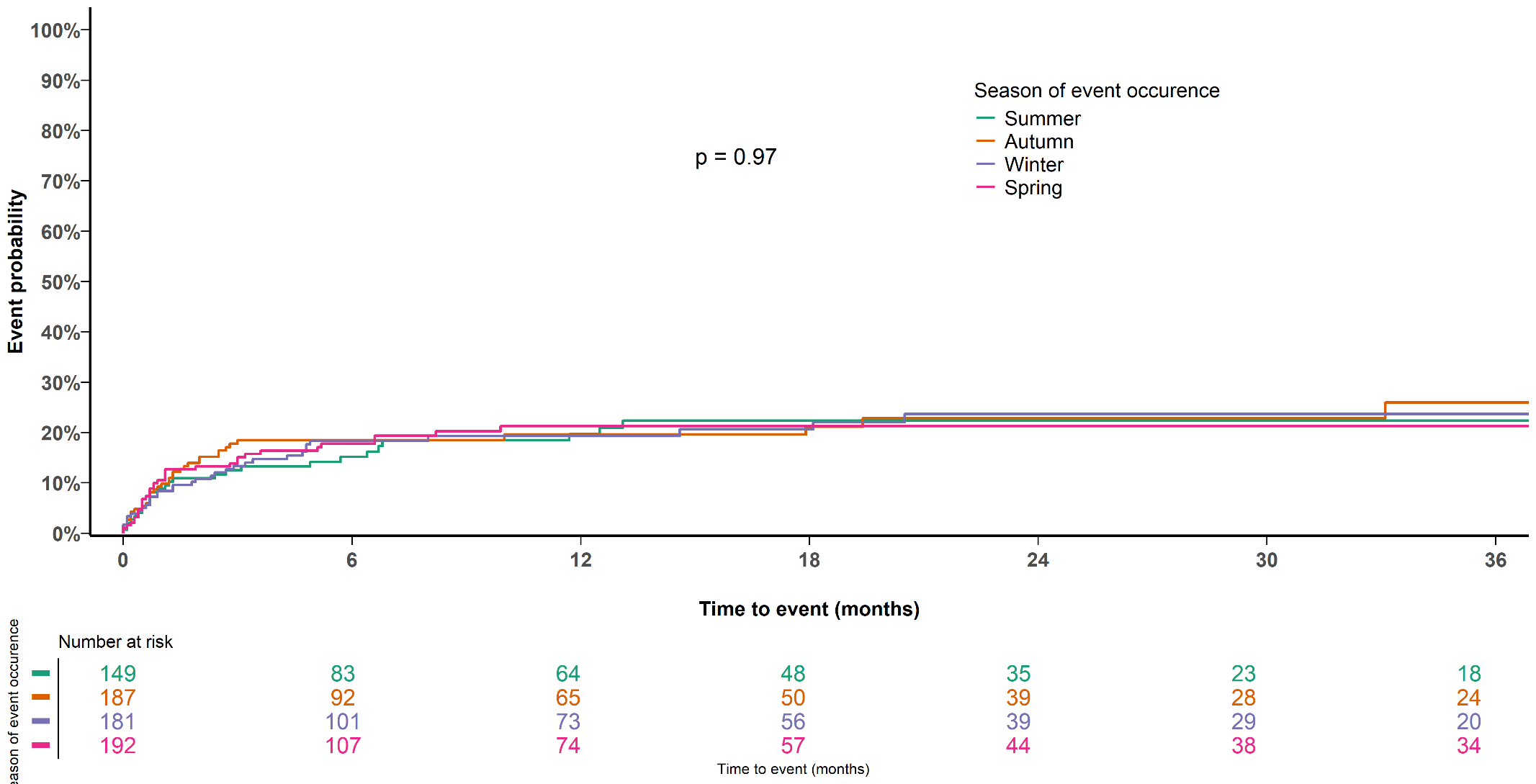


**Supplementary Figure 9A**: Kaplan-Meier curve of time to irAE stratified by season of irAE onset, for skin irAE (yes vs no).


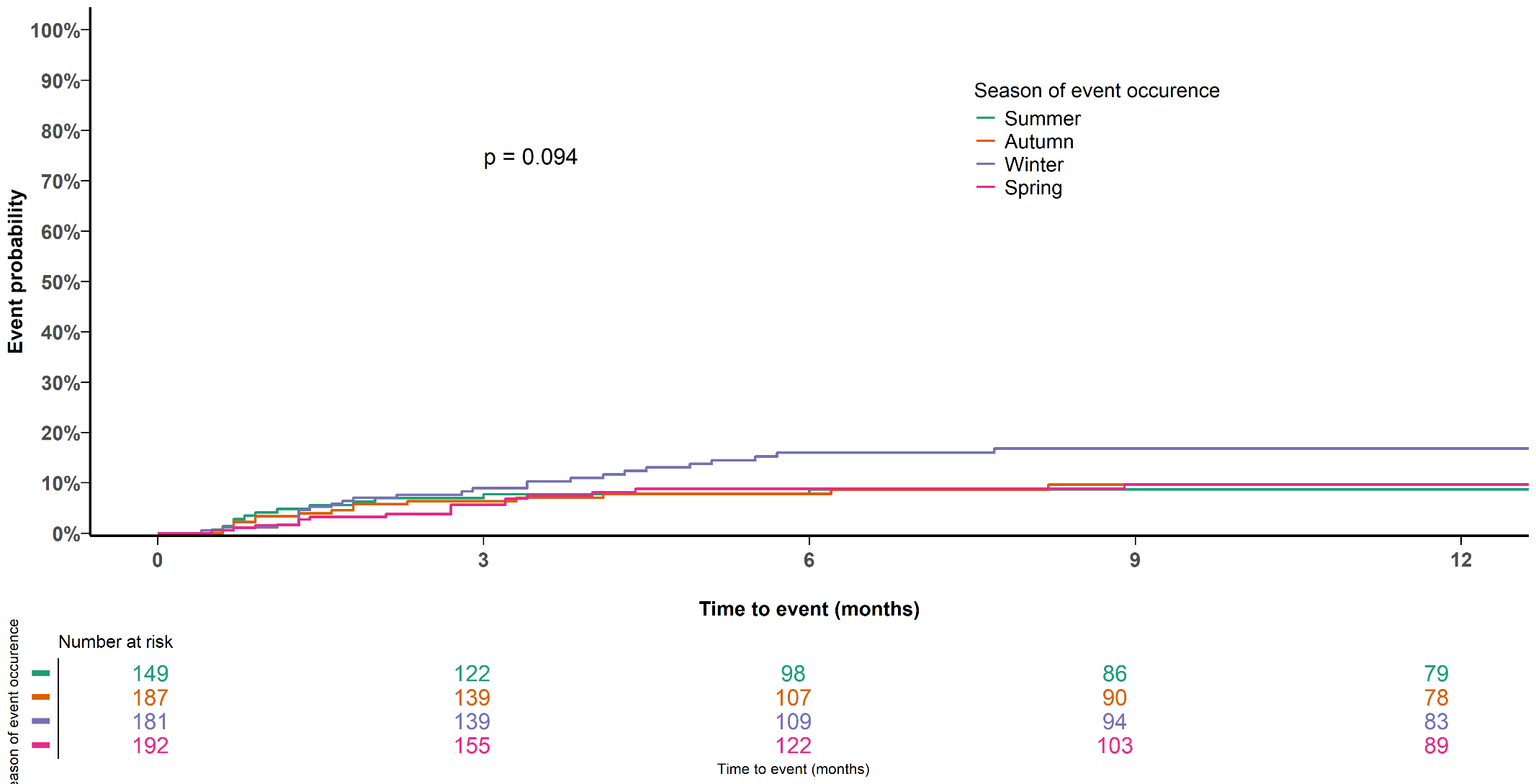


**Supplementary Figure 9B**: Kaplan-Meier curve of time to irAE stratified by season of irAE onset, for thyroiditis irAE (yes vs no).


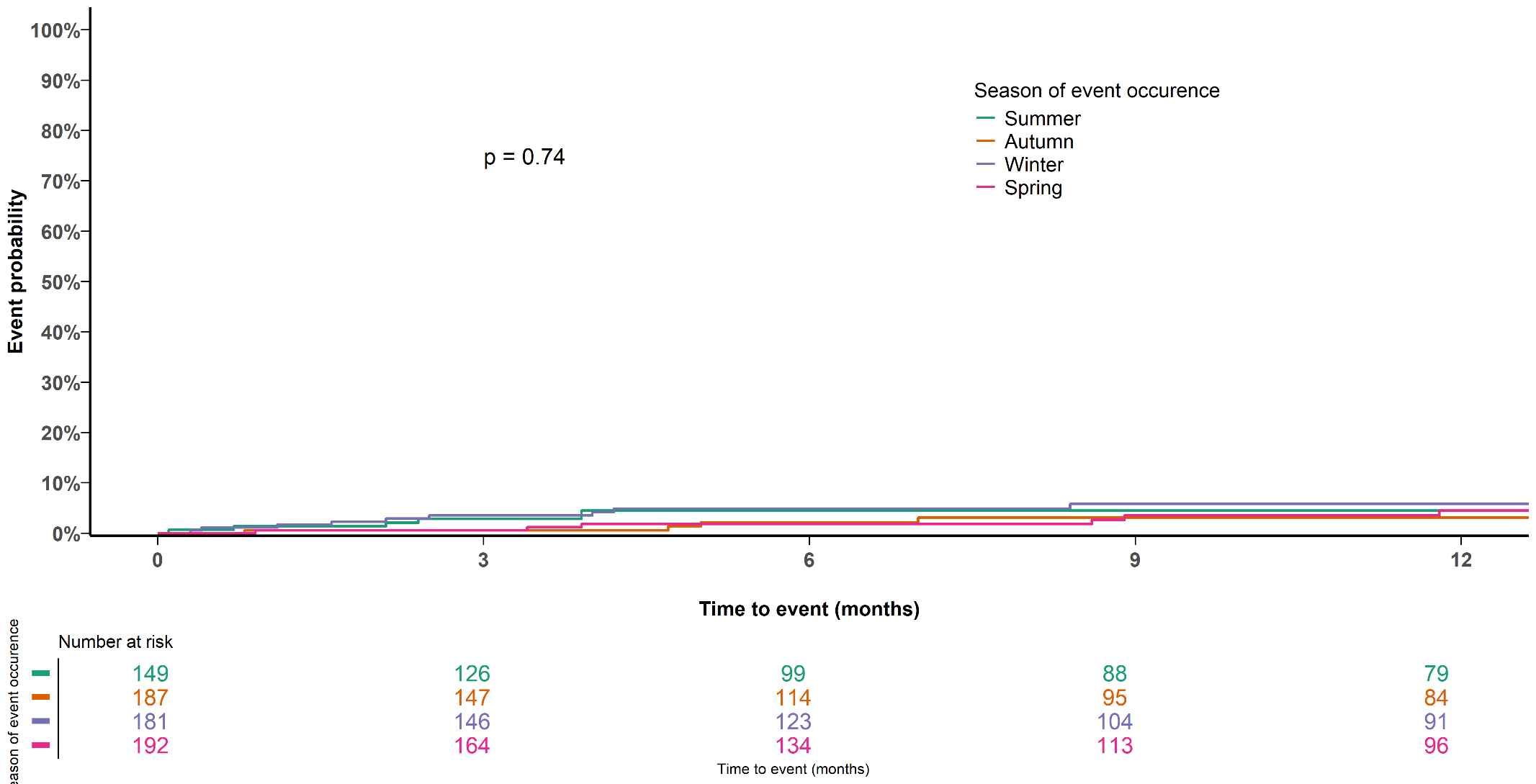


**Supplementary Figure 9C**: Kaplan-Meier curve of time to irAE stratified by season of irAE onset, for colitis irAE (yes vs no).


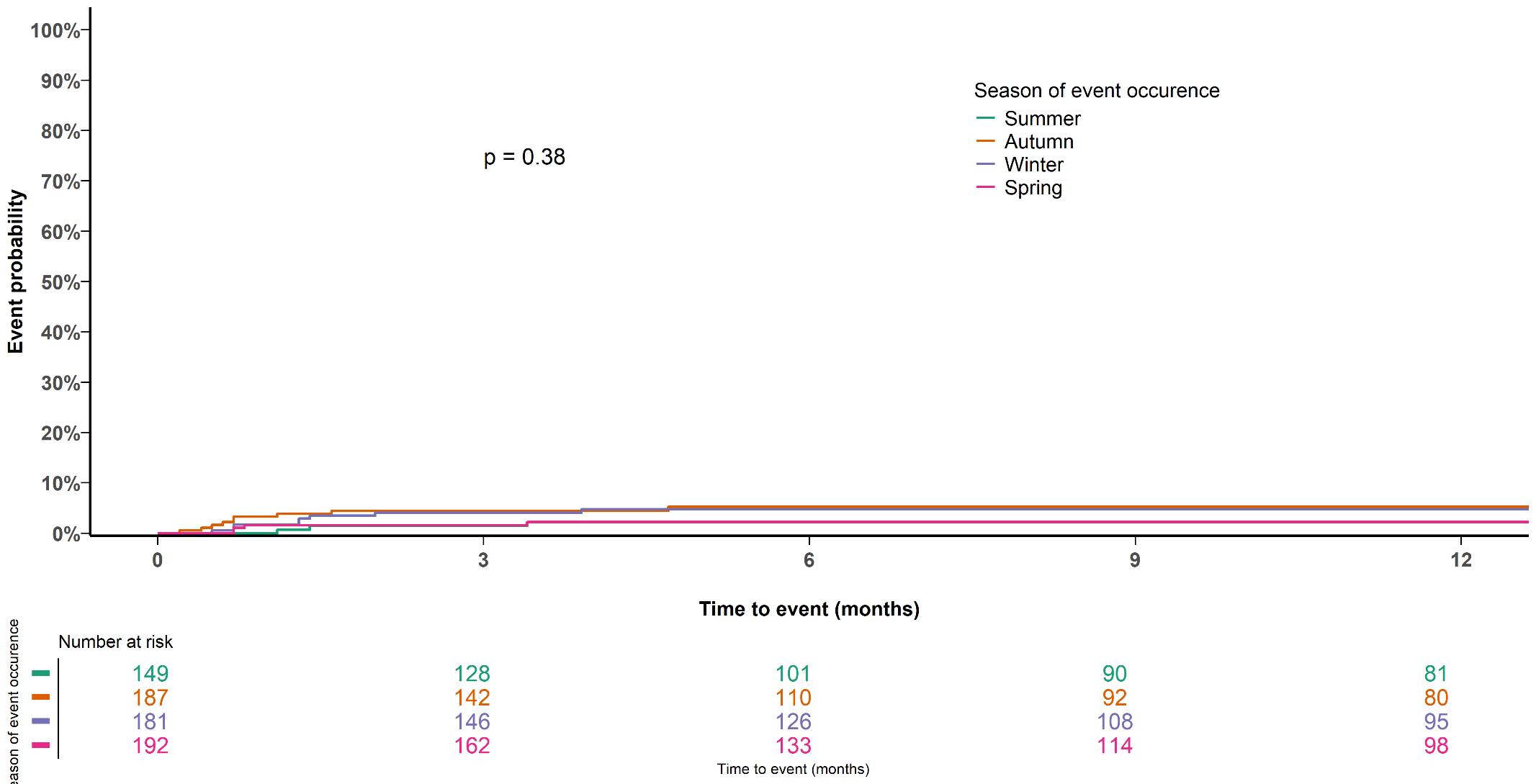


**Supplementary Figure 9D**: Kaplan-Meier curve of time to irAE stratified by season of irAE onset, for hepatitis irAE (yes vs no).


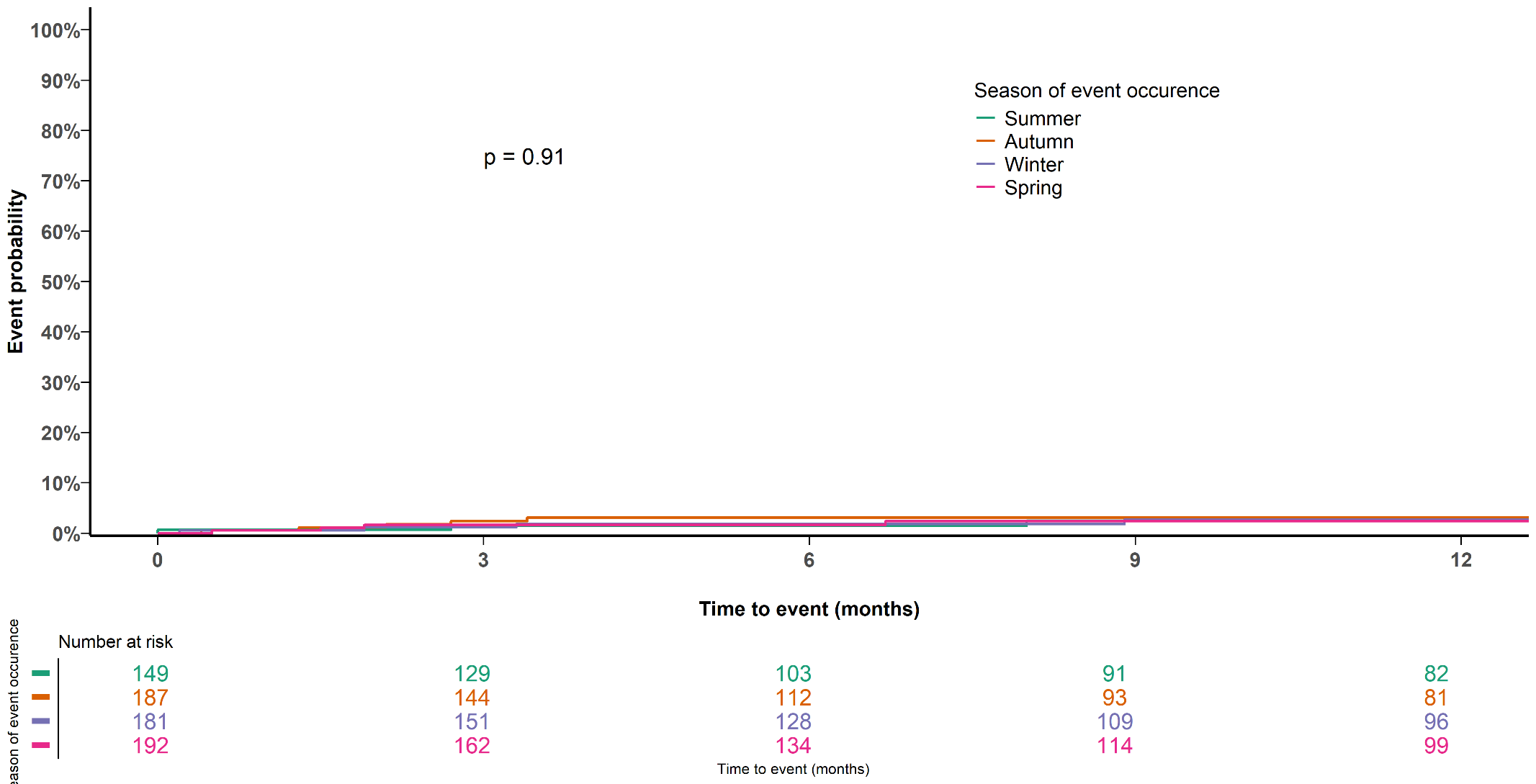


**Supplementary Figure 9E**: Kaplan-Meier curve of time to irAE stratified by season of irAE onset, for arthritis irAE (yes vs no).

**Supplementary Table 1**: All patients by tumour type and subsequent organ system grouping.

| **Tumour type** | **N = 709 (%)** | **Organ system group** |
| --- | --- | --- |
| Non-small cell lung cancer | 257 (36%) | Lung |
| Renal cell | 80 (11%) | Genitourinary |
| Endometrial cancer | 57 (8%) | Gynaecological |
| Urothelial | 44 (6%) | Genitourinary |
| Head & Neck | 44 (6%) | Head & Neck |
| Mesothelioma | 41 (6%) | Lung |
| Cholangiocarcinoma | 31 (4%) | Gastrointestinal |
| Cutaneous squamous cell carcinoma | 30 (4%) | Non-melanoma skin cancer |
| Colorectal | 26 (4%) | Gastrointestinal |
| Ovarian | 23 (3%) | Gynaecological |
| Unknown primary | 17 (2%) | Unknown primary |
| Gallbladder | 11 (2%) | Gastrointestinal |
| Gastro-oesophageal | 10 (1.4%) | Gastrointestinal |
| Small cell lung cancer | 7 (1.0%) | Lung |
| Merkel cell | 5 (0.7%) | Non-melanoma skin cancer |
| Nasopharyngeal | 5 (0.7%) | Head & Neck |
| Pancreatic | 5 (0.7%) | Gastrointestinal |
| Adrenocortical | 4 (0.6%) | Genitourinary |
| Cervical | 2 (0.3%) | Gynaecological |
| Penile | 2 (0.3%) | Genitourinary |
| Sarcoma | 2 (0.3%) | N/A |
| Small intestinal | 2 (0.3%) | Gastrointestinal |
| Thyroid | 1 (0.1%) | Head & Neck |
| Ampullary | 1 (0.1%) | Gastrointestinal |
| Gestational trophoblastic disease | 1 (0.1%) | Gynaecological |
| Vulval | 1 (0.1%) | Gynaecological |

**Supplementary Table 2**: Baseline table for irAEs, by 1, 2, or 3+ irAEs experienced.

| **Characteristics** | **All  (N = 339)** | **One irAE  (N = 216)** | **Two irAEs  (N = 86)** | **Three or more irAEs  (N = 37)** | **P-value** |
| --- | --- | --- | --- | --- | --- |
|  |  |  |  |  |  |
| **Number of ICI cycles until first irAE diagnosed** |  |  |  |  |  |
| Mean, SD | 5.5 (9.1) | 6.5 (9.5) | 4.4 (9.5) | 2.3 (1.6) | 0.017 |
| Median (range) | 2.0 (1.0, 73.0) | 3.0 (1.0, 71.0) | 2.0 (1.0, 73.0) | 2.0 (1.0, 6.0) |  |
|  |  |  |  |  |  |
| **Number of ICI cycles until first irAE diagnosed (categorised)** |  |  |  |  |  |
| 1 | 104 (30.7%) | 62 (59.6%) | 28 (26.9%) | 14 (13.5%) | 0.020 |
| 2 | 72 (21.2%) | 35 (48.6%) | 25 (34.7%) | 12 (16.7%) |  |
| 3 | 29 (8.6%) | 19 (65.5%) | 6 (20.7%) | 4 (13.8%) |  |
| 4 | 25 (7.4%) | 16 (64.0%) | 7 (28.0%) | 2 (8.0%) |  |
| 5+ | 109 (32.2%) | 84 (77.1%) | 20 (18.3%) | 5 (4.6%) |  |
|  |  |  |  |  |  |
| **Time in months to onset of first irAE** |  |  |  |  |  |
| Mean, SD | 3.8 (6.1) | 4.6 (6.9) | 2.6 (4.8) | 1.7 (1.7) | 0.004 |
| Median (range) | 1.6 (0.0, 48.8) | 2.0 (0.0, 48.8) | 1.3 (0.0, 33.3) | 1.3 (0.0, 8.0) |  |
|  |  |  |  |  |  |
| **Early (<1 month) vs late onset (>12 months) to first irAE** |  |  |  |  |  |
| Early | 120 (83.3%) | 69 (57.5%) | 35 (29.2%) | 16 (13.3%) | 0.017 |
| Late | 24 (16.7%) | 21 (87.5%) | 3 (12.5%) | 0 (0.0%) |  |
|  |  |  |  |  |  |
| **Highest irAE grade experienced** |  |  |  |  |  |
| 1 | 78 (23.0%) | 71 (91.0%) | 7 (9.0%) | 0 (0.0%) | <.001 |
| 2 | 136 (40.1%) | 82 (60.3%) | 42 (30.9%) | 12 (8.8%) |  |
| 3 | 111 (32.7%) | 55 (49.5%) | 32 (28.8%) | 24 (21.6%) |  |
| 4 | 12 (3.5%) | 7 (58.3%) | 4 (33.3%) | 1 (8.3%) |  |
| 5 | 2 (0.6%) | 1 (50.0%) | 1 (50.0%) | 0 (0.0%) |  |
|  |  |  |  |  |  |
| **Discontinued treatment due to irAE** |  |  |  |  |  |
| No | 238 (70.2%) | 162 (68.1%) | 58 (24.4%) | 18 (7.6%) |  |
| Yes | 101 (29.8%) | 54 (53.5%) | 28 (27.7%) | 19 (18.8%) | 0.004 |
|  |  |  |  |  |  |
| **Death by cause** |  |  |  |  |  |
| Disease | 159 (83.2%) | 111 (69.8%) | 36 (22.6%) | 12 (7.5%) | 0.571 |
| Immunotherapy-related | 2 (1.0%) | 1 (50.0%) | 1 (50.0%) | 0 (0.0%) |  |
| Complication from non-  immunotherapy treatment | 1 (0.5%) | 0 (0.0%) | 1 (100.0%) | 0 (0.0%) |  |
| Non-cancer related | 29 (15.2%) | 21 (72.4%) | 7 (24.1%) | 1(3.4%) |  |
|  |  |  |  |  |  |

**Supplementary Table 3**: Univariable and multivariable Cox regression for irAE (yes vs no).

|  | **Univariable** | | **Multivariable (†)** | |
| --- | --- | --- | --- | --- |
| **Variable** | **HR** | **P-value** | **HR** | **P-value** |
| **Season of immunotherapy start** |  |  |  |  |
| Autumn | 1 | 0.0735 | 1 | 0.1741 |
| Spring | 0.93 (0.69, 1.25) |  | 0.82 (0.60, 1.13) |  |
| Summer | 0.79 (0.57, 1.09) |  | 0.75 (0.53, 1.06) |  |
| Winter | 1.19 (0.89, 1.59) |  | 1.03 (0.76, 1.40) |  |
|  |  |  |  |  |
| **Sex** |  |  |  |  |
| Male | 1 | 0.0066 | 1 | 0.4574 |
| Female | 1.35 (1.09, 1.67) |  | 1.11 (0.84, 1.47) |  |
|  |  |  |  |  |
| **Age (years) at cycle 1 of immunotherapy** | 0.99 (0.98, 1.00) | 0.0985 | 1.01 (1.00, 1.02) | 0.0751 |
|  |  |  |  |  |
| **Age category** |  |  |  |  |
| Less than 45 | 1 | 0.2196 |  |  |
| 45 to 54 | 0.63 (0.38, 1.06) |  |  |  |
| 55 to 65 | 0.63 (0.39, 1.00) |  |  |  |
| 65 to 74 | 0.65 (0.42, 1.02) |  |  |  |
| Over 75 | 0.57 (0.36, 0.91) |  |  |  |
|  |  |  |  |  |
| **ECOG** |  |  |  |  |
| 0 | 1 | <.0001 | 1 | 0.0166 |
| 1 | 0.64 (0.51, 0.81) |  | 0.77 (0.60, 0.99) |  |
| 2 | 0.38 (0.25, 0.57) |  | 0.53 (0.34, 0.82) |  |
| 3 | 0.41 (0.13, 1.28) |  | 0.36 (0.09, 1.48) |  |
|  |  |  |  |  |
| **Smoking history** |  |  |  |  |
| Yes | 1 | 0.0045 | 1 | 0.7849 |
| No | 1.38 (1.10, 1.72) |  | 0.96 (0.75, 1.25) |  |
|  |  |  |  |  |
| **Cancer type** |  |  |  |  |
| Gastrointestinal | 1 | <.0001 | 1 | 0.2056 |
| Genitourinary | 0.82 (0.57, 1.18) |  | 0.84 (0.56, 1.28) |  |
| Gynaecological | 1.55 (1.06, 2.27) |  | 1.16 (0.74, 1.82) |  |
| Head & Neck | 0.44 (0.24, 0.79) |  | 0.59 (0.31, 1.10) |  |
| Lung | 0.50 (0.36, 0.70) |  | 0.66 (0.45, 0.97) |  |
| Non-melanoma skin cancer | 0.47 (0.26, 0.85) |  | 0.62 (0.31, 1.22) |  |
| Unknown primary | 0.66 (0.28, 1.54) |  | 0.87 (0.36, 2.08) |  |
|  |  |  |  |  |
| **Treatment length** | 1.00 (0.99, 1.01) | 0.7918 | 1.00 (0.99, 1.01) | 0.9208 |
|  |  |  |  |  |
| **Previous systemic therapy** |  |  |  |  |
| No | 1 | 0.2866 | 1 | 0.7286 |
| Yes | 0.89 (0.72, 1.10) |  | 0.96 (0.74, 1.23) |  |
|  |  |  |  |  |
| **Number of ICI** |  |  |  |  |
| Single ICI | 1 |  | 1 | <.0001 |
| Doublet ICI | 2.73 (2.20, 3.39) | <.0001 | 1.96 (1.46, 2.63) |  |

*† Where there are two variables with the same information, the variable with the lowest p-value for the univariable regression is included in the multivariate regression.*

**Supplementary Table 4**: Multivariable Cox regression for irAE (yes vs no), full cohort vs lung cancer patients only.

|  | **All patients** | | **Lung cancer only** | |
| --- | --- | --- | --- | --- |
| **Variable** | **HR** | **P-value** | **HR** | **P-value** |
| **Season of immunotherapy start** |  |  |  |  |
| Autumn | 1 | 0.1741 | 1 | 0.5376 |
| Spring | 0.82 (0.60, 1.13) |  | 0.98 (0.52, 1.82) |  |
| Summer | 0.75 (0.53, 1.06) |  | 0.64 (0.31, 1.29) |  |
| Winter | 1.03 (0.76, 1.40) |  | 0.70 (0.35, 1.40) |  |
| **Sex** |  |  |  |  |
| Male | 1 | 0.4574 | 1 | 0.1128 |
| Female | 1.11 (0.84, 1.47) |  | 0.62 (0.34, 1.12) |  |
|  |  |  |  |  |
| **Age (years) at cycle 1 of immunotherapy** | 1.01 (1.00, 1.02) | 0.0751 | 0.98 (0.95, 1.01) | 0.2755 |
| **Age category** |  |  |  |  |
| Less than 45 |  |  |  |  |
| 45 to 54 |  |  |  |  |
| 55 to 65 |  |  |  |  |
| 65 to 74 |  |  |  |  |
| Over 75 |  |  |  |  |
| **ECOG** |  |  |  |  |
| 0 | 1 | 0.0166 | 1 | 0.4587 |
| 1 | 0.77 (0.60, 0.99) |  | 0.70 (0.39, 1.24) |  |
| 2 | 0.53 (0.34, 0.82) |  | 0.86 (0.39, 1.90) |  |
| 3 | 0.36 (0.09, 1.48) |  | 1.00 (1.00, 1.00) |  |
| **Smoking history** |  |  |  |  |
| Yes | 1 | 0.7849 | 1 | 0.3037 |
| No | 0.96 (0.75, 1.25) |  | 1.47 (0.70, 3.07) |  |
| **Cancer type** |  |  |  |  |
| Gastrointestinal | 1 | 0.2056 |  |  |
| Genitourinary | 0.84 (0.56, 1.28) |  |  |  |
| Gynaecological | 1.16 (0.74, 1.82) |  |  |  |
| Head & Neck | 0.59 (0.31, 1.10) |  |  |  |
| Lung | 0.66 (0.45, 0.97) |  |  |  |
| Non-mel skin cancer | 0.62 (0.31, 1.22) |  |  |  |
| Unknown primary | 0.87 (0.36, 2.08) |  |  |  |
|  |  |  |  |  |
| **Treatment length** | 1.00 (0.99, 1.01) | 0.9208 | 0.91 (0.88, 0.94) | <.0001 |
| **Previous systemic therapy** |  |  |  |  |
| No | 1 | 0.7286 | 1 | 0.1188 |
| Yes | 0.96 (0.74, 1.23) |  | 1.47 (0.91, 2.37) |  |
|  |  |  |  |  |
| **Number of ICI** |  |  |  |  |
| Single ICI | 1 | <.0001 | 1 | 0.3316 |
| Doublet ICI | 1.96 (1.46, 2.63) |  | 0.71 (0.36, 1.41) |  |
